# Correction: Strong Selection at MHC in Mexicans since Admixture

**DOI:** 10.1371/journal.pgen.1005983

**Published:** 2016-04-01

**Authors:** The PLOS Genetics Staff

## Notice of Republication

This article was republished on March 16, 2016, to remove instances of the word ‘black’ that appeared throughout the text as a result of a LaTeX file conversion error. Please download this article again to view the correct version. The originally published, uncorrected article and the republished, corrected articles are provided here for reference.

## Supporting Information

S1 File Originally published, uncorrected article. (PDF)

S2 File Republished, corrected article. (PDF)
