# Supplementary material for: Strong Selection at MHC in Mexicans since Admixture
Source: PLoS Genet. 2016 Feb 10;12(2):e1005847. doi: 10.1371/journal.pgen.1005847 (PMC4749250; doi:10.1371/journal.pgen.1005847)
Supplement: S1 Table — ALL means CEU+TSI−YRI+MKK−MAYA, and ssd means sample standard deviation. (PDF) [file pgen.1005847.s001.pdf]

Supporting Information

Strong Selection at MHC in Mexicans since Admixture. Q. Zhou, L. Zhao, Y. Guan.
PLoS Genetics. 2016

Training set	Ancestry	ssd	Min	Mean	Max
CEU–YRI–MAYA	African	0.017	0.047	0.079	0.325
	Amerindian	0.034	0.941	1.104	1.258
	European	0.033	0.676	0.818	0.967
CEU–MKK–MAYA	African	0.018	0.056	0.095	0.354
	Amerindian	0.033	0.934	1.109	1.236
	European	0.032	0.659	0.796	0.960
TSI–YRI–MAYA	African	0.013	0.046	0.076	0.263
	Amerindian	0.032	0.954	1.116	1.250
	European	0.031	0.680	0.808	0.953
TSI–MKK–MAYA	African	0.014	0.053	0.088	0.260
	Amerindian	0.032	0.937	1.118	1.260
	European	0.030	0.670	0.794	0.939
ALL	African	0.016	0.050	0.083	0.297
	Amerindian	0.033	0.933	1.102	1.224
	European	0.031	0.708	0.815	0.949

Table S1: Summary statistics for different sets of training samples for autosomes of the Lipid dataset. ALL means CEU+TSI–YRI+MKK–MAYA, and ssd means sample standard deviation.
