# Supplementary material for: Strong Selection at MHC in Mexicans since Admixture
Source: PLoS Genet. 2016 Feb 10;12(2):e1005847. doi: 10.1371/journal.pgen.1005847 (PMC4749250; doi:10.1371/journal.pgen.1005847)
Supplement: S1 Fig — A) Average dosages for Amerindian (blue), European (red), and African (green) ancestries for Viva (top) and Lipid (bottom) datasets with training samples of CEU−YRI−MAYA. B) Average dosages for Amerindian (blue), European (red), and African (green) ancestries for Lipid dataset with training samples of CEU−MKK−MAYA (top) and TSI−MKK−MAYA (bottom). C)Average dosages for Amerindian (blue), European (red), and African (green) ancestries for Lipid dataset with training samples of TSI−YRI−MAYA (top) and CEU+TSI−YRI+MKK−MAYA (bottom). (PDF) [file pgen.1005847.s002.pdf]

Supporting Information

Strong Selection at MHC in Mexicans since Admixture. Q. Zhou, L. Zhao, Y. Guan. PLoS Genetics. 2016

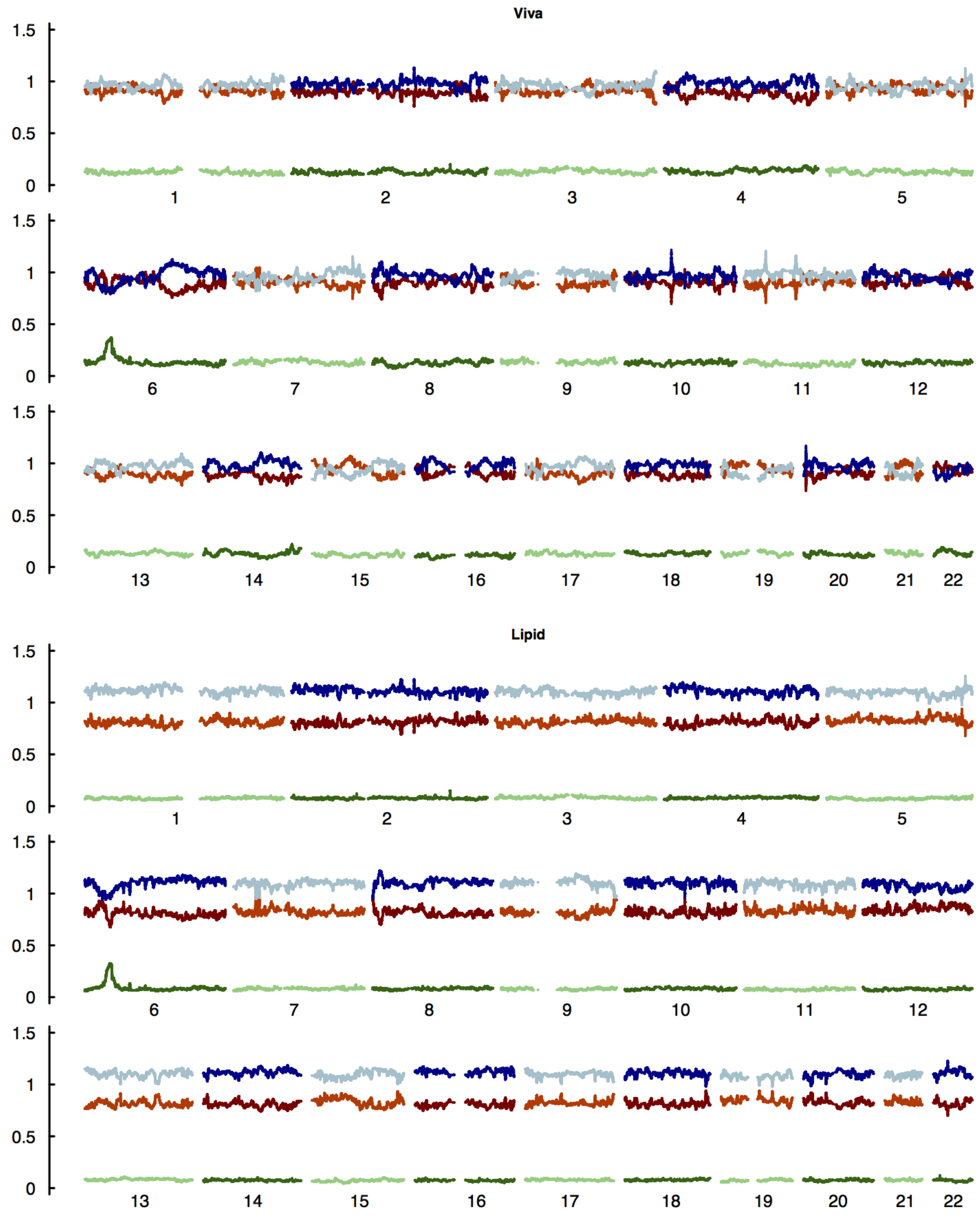

Figure S1A Average dosages for Amerindian (blue), European (red), and African (green) ancestries for Viva and Lipid datasets with training samples of CEU+YRI+Maya. Plot shows all 22 autosomes.

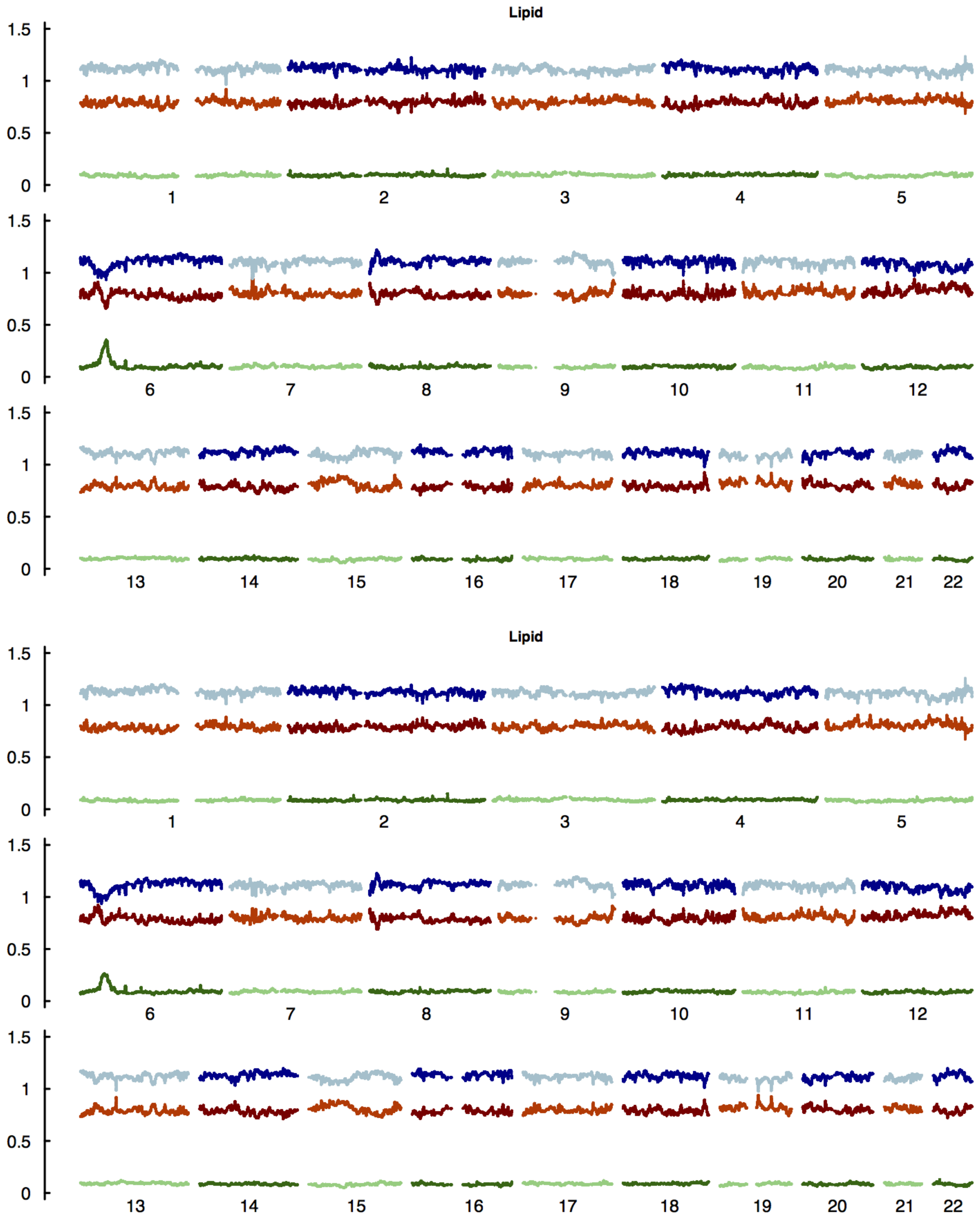

Figure S1B Average dosages for Amerindian (blue), European (red), and African (green) ancestries for Lipid datasets with training samples of CEU+MKK+Maya, and TSI+MKK+Maya.

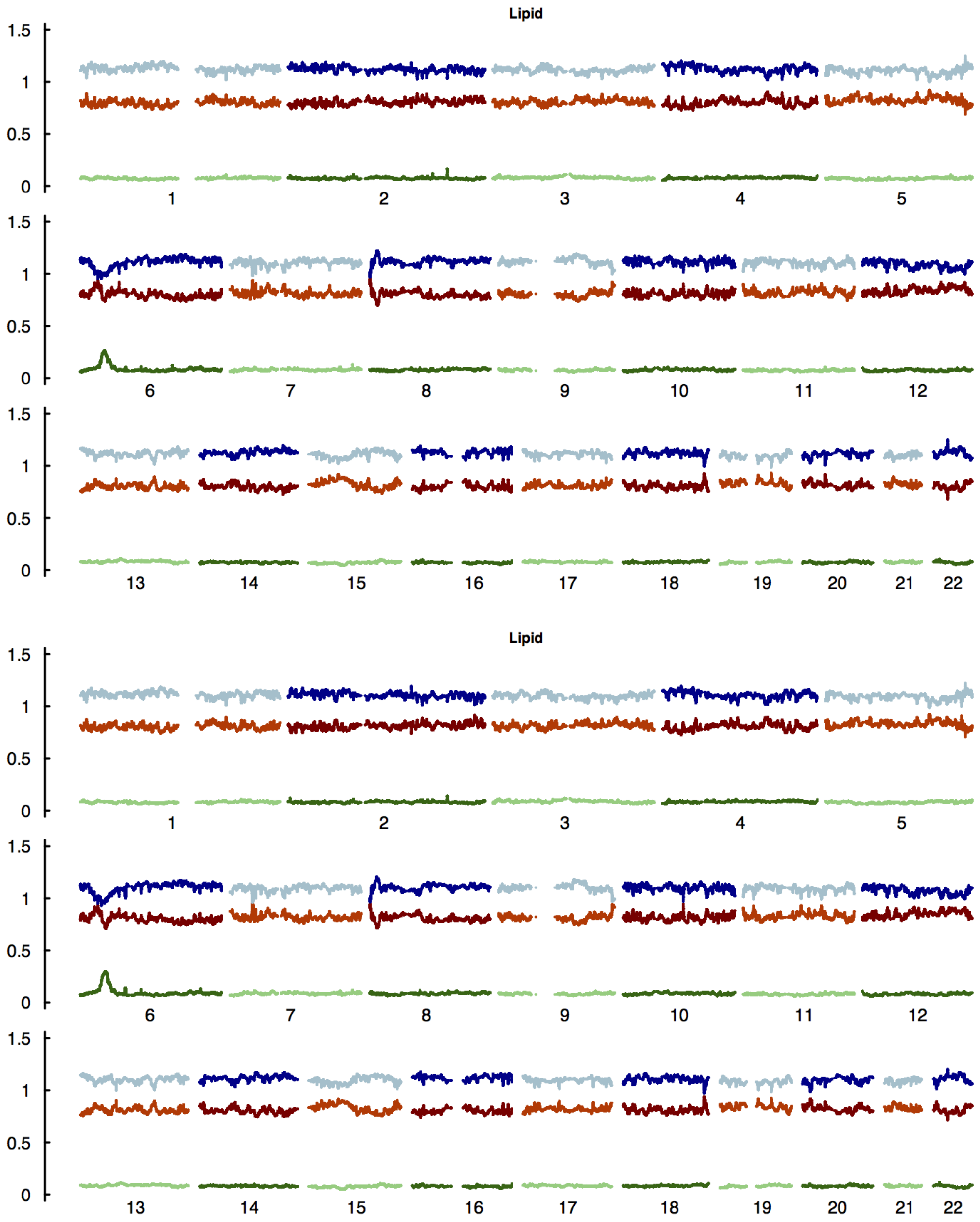

Figure S1C Average dosages for Amerindian (blue), European (red), and African (green) ancestries for Lipid datasets with training samples of TSI+YRI+Maya, and CEU-TSI+YRI-MKK+Maya.
