# Supplementary material for: Strong Selection at MHC in Mexicans since Admixture
Source: PLoS Genet. 2016 Feb 10;12(2):e1005847. doi: 10.1371/journal.pgen.1005847 (PMC4749250; doi:10.1371/journal.pgen.1005847)
Supplement: S2 Fig — The five sets of training samples are shown in the legend, where ALL means CEU+TSI−YRI+MKK−MAYA. The comparison was performed with chromosome 6 of Lipid dataset. (PDF) [file pgen.1005847.s003.pdf]

## Supporting Information

**Strong Selection at MHC in Mexicans since Admixture.** Q. Zhou, L. Zhao, Y. Guan.
PLoS Genetics. 2016

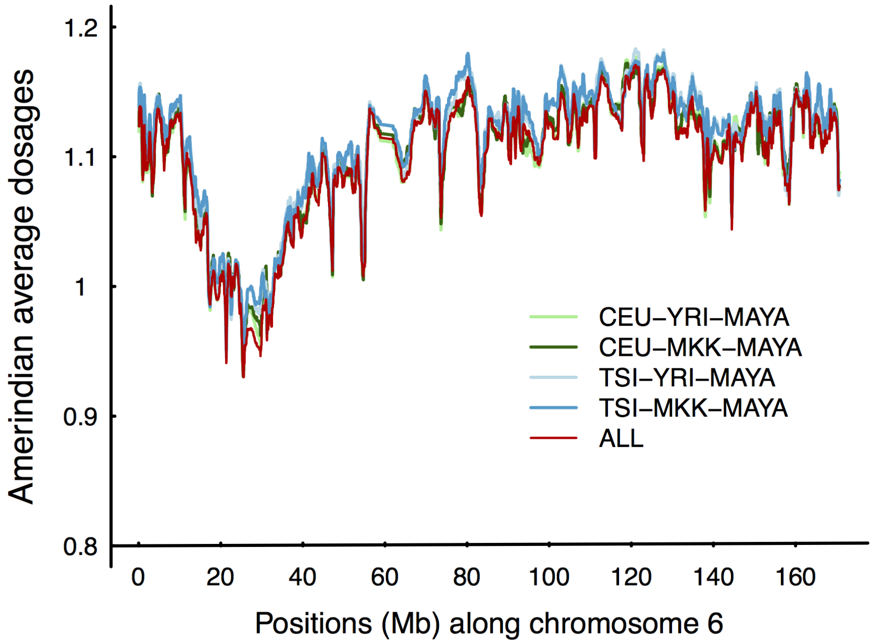


**Fig S2.** Comparison of Amerindian ancestry dosages for five sets of training samples shown in the legend, where ALL means CEU+TSI–YRI+MKK–MAYA. The comparison was performed with chromosome 6 of Lipid dataset.
