# Supplementary material for: Strong Selection at MHC in Mexicans since Admixture
Source: PLoS Genet. 2016 Feb 10;12(2):e1005847. doi: 10.1371/journal.pgen.1005847 (PMC4749250; doi:10.1371/journal.pgen.1005847)
Supplement: S3 Fig — Bayes factors (BF) were computed using BIMBAM. The horizontal blue line is log10 BF = 10. (PDF) [file pgen.1005847.s004.pdf]

Supporting Information

Strong Selection at MHC in Mexicans since Admixture. Q. Zhou, L. Zhao, Y. Guan.
PLoS Genetics. 2016

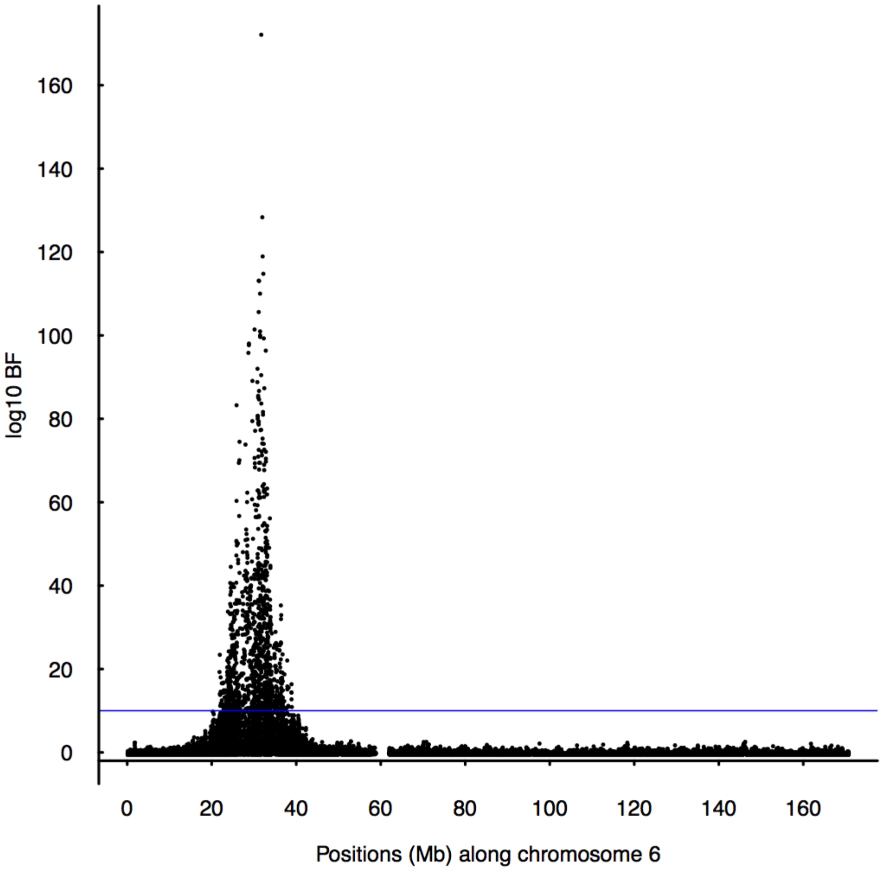

Fig S3. SNP associated with enrichment of African local ancestry in Mexican lipid study. Bayes factors (BF) were computed using BIMBAM. The horizontal blue line is $\log_{10} BF = 10$.
