# Supplementary material for: Strong Selection at MHC in Mexicans since Admixture
Source: PLoS Genet. 2016 Feb 10;12(2):e1005847. doi: 10.1371/journal.pgen.1005847 (PMC4749250; doi:10.1371/journal.pgen.1005847)
Supplement: S4 Fig — A) Comparison between LAMP-LD, RFMix, and ELAI under different simulation conditions, Part I. There are 9 combinations of crossover probability (0.1,0.2, and 0.5) and selection coefficients (0.02,0.05, and 0.10) for two sizes of the mid-section. This plot is for mid-section of size 1 Mb. The mid-section harbors alleles under selection, and a smaller size produces a more challenging problem. Plots also compare effects of phasing errors (2% for cohort and the Amerindian training sample and 1% for the other two training samples). RFMix underperformed after phasing errors were introduced in (b). Compared to RFMix, LAMP-LD was less sensitive to phasing errors. ELAI was unaffected by phasing errors. Parameters for LAMP-LD: window size = 100, number of HMM states = 25; for RFMix: window size = 0.1 cM, which approximately contains 100 SNPs. Both parameter settings were used by the 1000 Genomes admixture analysis group. B) Comparison between LAMP-LD, RFMix, and ELAI under different simulation conditions, Part II. The same simulation setup as in S4A Fig but with mid-section of size 0.5 Mb. C) RFMix performance with different switch-errors. This is the same dataset as used in S4B Fig. In the legends, the number before the plus sign is the switch-error for cohort and the Amerindian training sample, and the number after is for the European and African training samples. D) RFMix performance with different choices of window size. The mean absolute deviation was computed from the same dataset that was used in S4B Fig and averaged over 9 simulation parameter settings (recombination probability and selection strength). The switch-errors were 2% for cohort and the Amerindian training sample and 1% for the other two training samples. We used centi-Morgan (cM) to measure the window size, which is the unit used by RFMix. In our simulations, 1 cM contains roughly 1000 SNPs. E) RFMix performs well with the optimal window size. This is the same dataset as used in S4B Fig. When the optimal w [file pgen.1005847.s005.pdf]

Supporting Information

Strong Selection at MHC in Mexicans since Admixture. Q. Zhou, L. Zhao, Y. Guan. PLoS Genetics. 2016

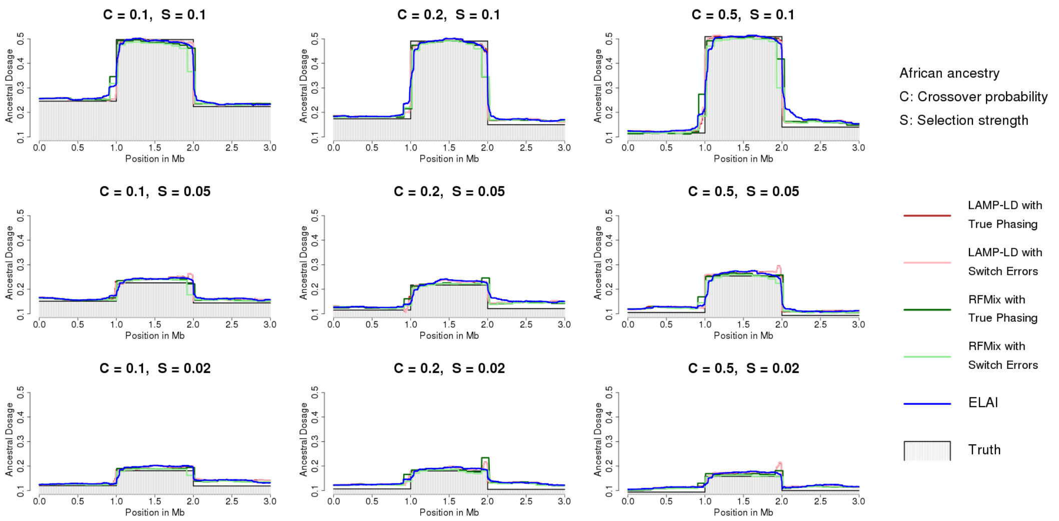

Fig S4A: Comparison between LAMP-LD, RFMix, and ELAI under different simulation conditions, Part I There are 9 combinations of crossover probability $(0.1, 0.2,$ and $0.5)$ and selection coefficients $(0.02, 0.05,$ and $0.10)$ for two sizes of the mid-section. This plot is for mid-section of size 1 Mb. The mid-section harbors alleles under selection, and a smaller size produces a more challenging problem. Plots also compare effects of phasing errors (2% for cohort and the Amerindian training sample and 1% for the other two training samples). RFMix underperformed after phasing errors were introduced in (b). Compared to RFMix, LAMP-LD was less sensitive to phasing errors. ELAI was unaffected by phasing errors. Parameters for LAMP-LD: window size $= 100$, number of HMM states $= 25$; for RFMix: window size $= 0.1$ cM, which approximately contains 100 SNPs. Both parameter settings were used by the 1000 Genomes admixture analysis group.

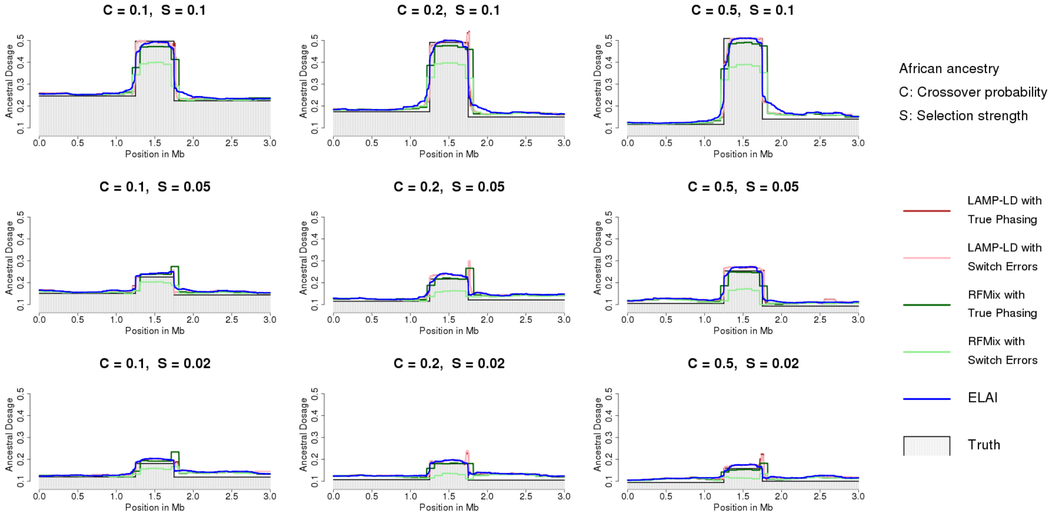

Fig S4B: Comparison between LAMP-LD, RFMix, and ELAI under different simulation conditions, Part II. The same simulation setup as in Fig S4A but with mid-section of size 0.5 Mb.

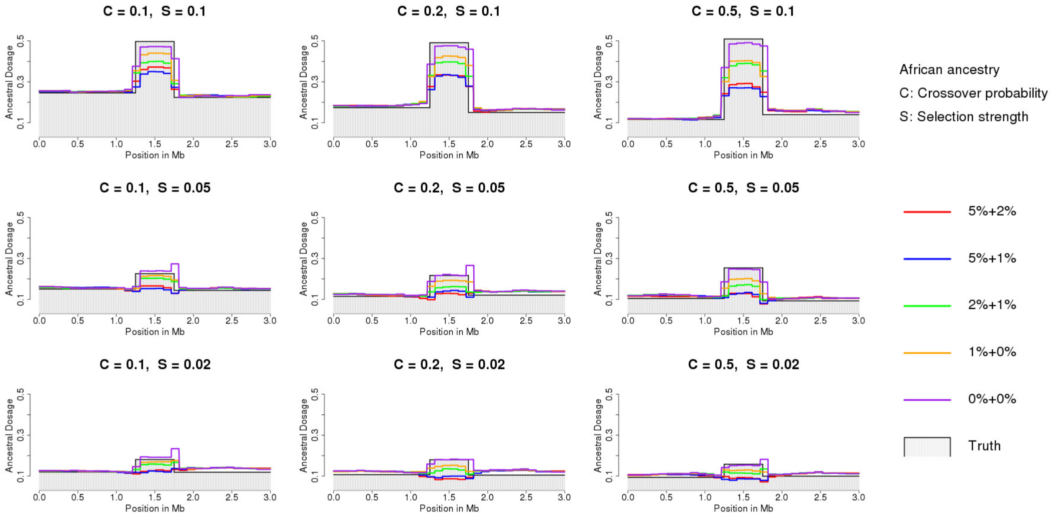

Fig S4C: RFMix performance with different switch-errors. This is the same dataset as used in Fig S4B. In the legends, the number before the plus sign is the switch-error for cohort and the Amerindian training sample, and the number after is for the European and African training samples.

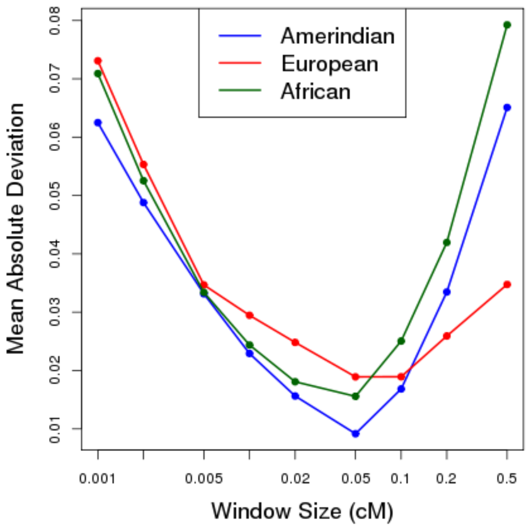

Fig S4D: RFMix performance with different choices of window size. The mean absolute deviation was computed from the same dataset that was used in Fig S4B and averaged over 9 simulation parameter settings (recombination probability and selection strength). The switch-errors were 2% for cohort and the Amerindian training sample and 1% for the other two training samples. We used centi-Morgan (cM) to measure the window size, which is the unit used by RFMix. In our simulations, 1 cM contains roughly 1000 SNPs.

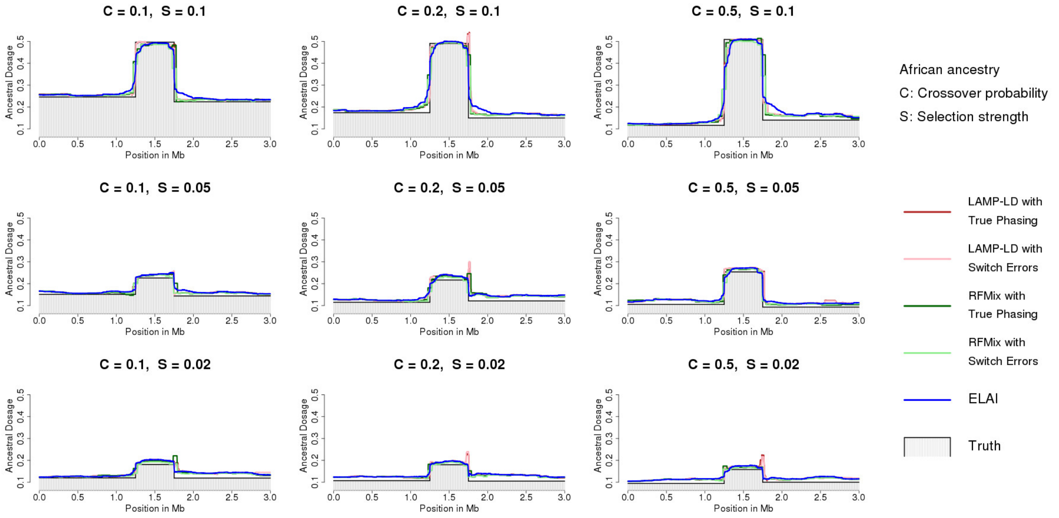

Fig S4E: RFMix performs well with the optimal window size. This is the same dataset as used in Fig S4B. When the optimal window size is used, RFMix performs well in the presence of phasing errors (2% for cohort and the Amerindian training sample and 1% for the other two training samples).
