# Supplementary material for: Strong Selection at MHC in Mexicans since Admixture
Source: PLoS Genet. 2016 Feb 10;12(2):e1005847. doi: 10.1371/journal.pgen.1005847 (PMC4749250; doi:10.1371/journal.pgen.1005847)
Supplement: S5 Fig — LAMP-LD (first column) and RFMix (second column) discovered excessive African average dosages at MHC for both Viva (first row) and Lipid (second row) datasets. (PDF) [file pgen.1005847.s006.pdf]

Supporting Information

Strong Selection at MHC in Mexicans since Admixture. Q. Zhou, L. Zhao, Y. Guan. PLoS Genetics. 2016

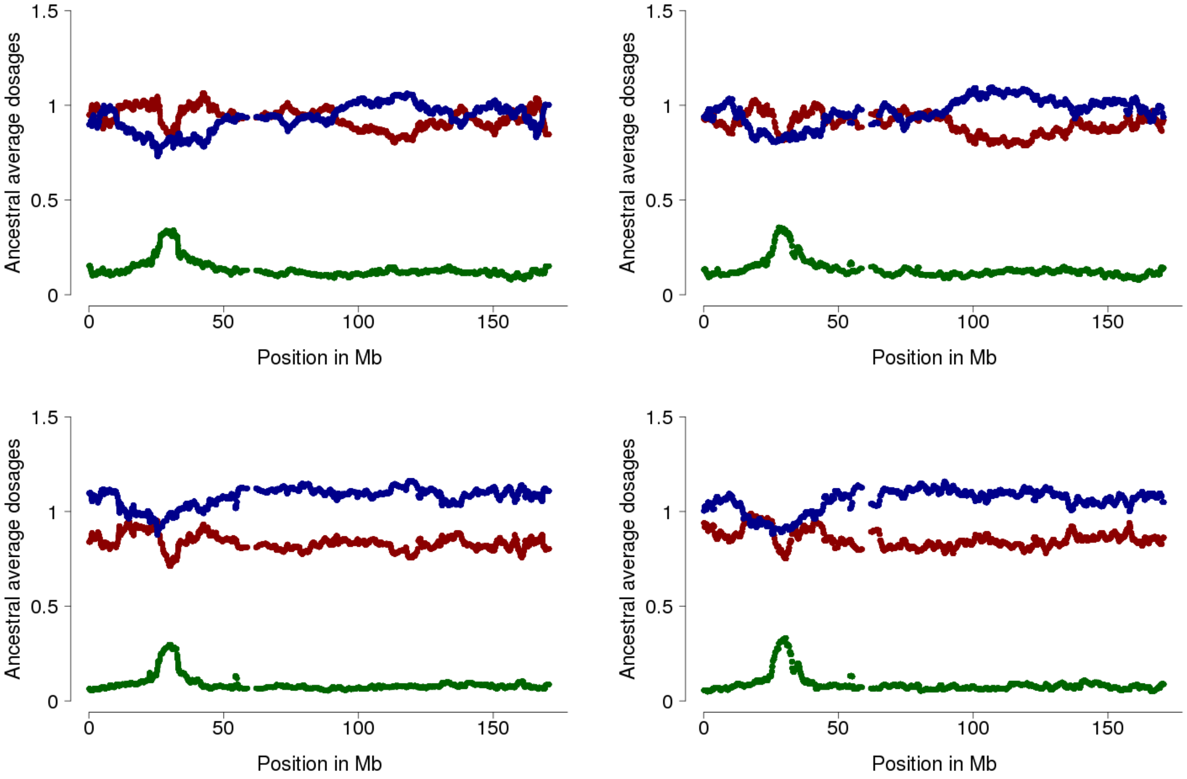

Fig S5. LAMP-LD (first column) and RFMix (second column) discovered excessive peak at MHC in both Viva (first row) and Lipid (second row) data.
