# Supplementary material for: Strong Selection at MHC in Mexicans since Admixture
Source: PLoS Genet. 2016 Feb 10;12(2):e1005847. doi: 10.1371/journal.pgen.1005847 (PMC4749250; doi:10.1371/journal.pgen.1005847)
Supplement: S6 Fig — The grey lines indicate the truth. The black lines are the inferred African average dosages by ELAI with only European and African training samples. There are 9 combinations of crossover probability (0.1,0.2, and 0.5) and selection coefficients (0.02,0.05, and 0.10), and the size of the mid-section is 0.5 Mb. On each plot the main text displays the simulation parameters with C for crossover probability and S for selection coefficient. For example, C = 0.2, S = 0.05 means crossover probability is 0.2 and selection coefficient is 0.05. (PDF) [file pgen.1005847.s007.pdf]

Supporting Information

Strong Selection at MHC in Mexicans since Admixture. Q. Zhou, L. Zhao, Y. Guan. PLoS Genetics. 2016

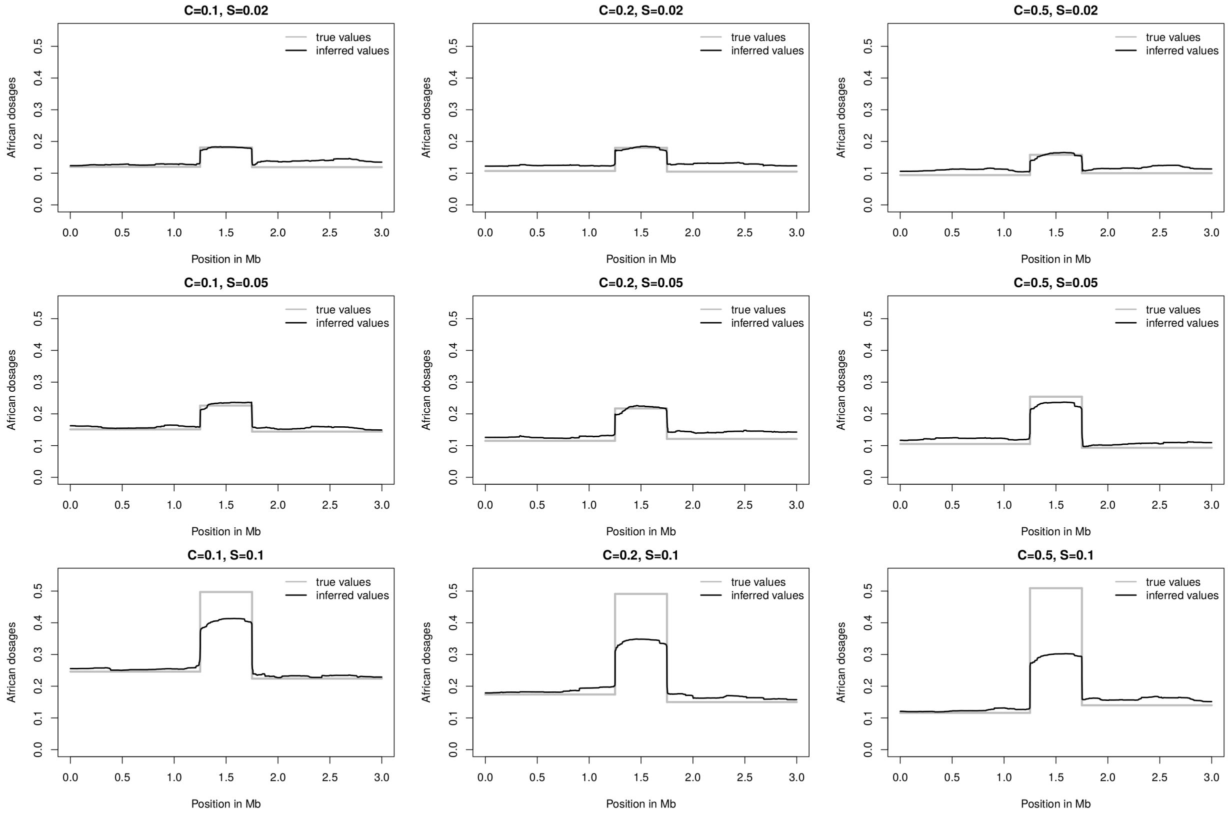

Fig S6. Simulation studies to exclude Amerindian training samples. The grey lines indicate the truth. The black lines are the inferred African average dosages by ELAI with only European and African training samples. There are 9 combinations of crossover probability $(0.1, 0.2,$ and $0.5)$ and selection coefficients $(0.02, 0.05,$ and $0.10)$, and the size of the mid-section is 0.5 Mb. On each plot the main text displays the simulation parameters with C for crossover probability and S for selection coefficient. For example, $C = 0.2, S = 0.05$ means crossover probability is 0.2 and selection coefficient is 0.05.
