# Supplementary material for: Correction: Strong Selection at MHC in Mexicans since Admixture
Source: PLoS Genet. 2016 Apr 1;12(4):e1005983. doi: 10.1371/journal.pgen.1005983 (PMC4817986; doi:10.1371/journal.pgen.1005983)
Supplement: S2 File — (PDF) [file pgen.1005983.s002.pdf]

PLOS | GENETICS

RESEARCH ARTICLE

Strong Selection at MHC in Mexicans since Admixture

Quan Zhou[1,2,3], **Liang Zhao**[1,2], **Yongtao Guan**[1,2,3,4]*

1 USDA/ARS Children's Nutrition Research Center, Houston, Texas, United States of America,
2 Department of Pediatrics, Baylor College of Medicine, Houston, Texas, United States of America,
3 Program of Structure and Computational Biology and Molecular Biophysics, Baylor College of Medicine, Houston, Texas, United States of America, **4** Department of Molecular and Human Genetics, Baylor College of Medicine, Houston, Texas, United States of America

* yongtaog@bcm.edu

CrossMark
click for updates

OPEN ACCESS

Citation: Zhou Q, Zhao L, Guan Y (2016) Strong Selection at MHC in Mexicans since Admixture. PLoS Genet 12(2): e1005847. doi:10.1371/journal.pgen.1005847

Editor: Joshua M. Akey, University of Washington, UNITED STATES

Received: July 6, 2015

Accepted: January 14, 2016

Published: February 10, 2016

Copyright: © 2016 Zhou et al. This is an open access article distributed under the terms of the Creative Commons Attribution License, which permits unrestricted use, distribution, and reproduction in any medium, provided the original author and source are credited.

Data Availability Statement: All datasets are available from the Database of Genotypes and Phenotypes (dbGaP, accession numbers phs000616.v1.p1, phs000618.v1.p1).

Funding: This project is funded by United States Department of Agriculture/Agricultural Research Service USDA/ARS 6250-51000-057 and National Institutes of Health R01HG008157. The funders had no role in study design, data collection and analysis, decision to publish, or preparation of the manuscript.

Competing Interests: The authors have declared that no competing interests exist.

Abstract

Mexicans are a recent admixture of Amerindians, Europeans, and Africans. We performed local ancestry analysis of Mexican samples from two genome-wide association studies obtained from dbGaP, and discovered that at the MHC region Mexicans have excessive African ancestral alleles compared to the rest of the genome, which is the hallmark of recent selection for admixed samples. The estimated selection coefficients are 0.05 and 0.07 for two datasets, which put our finding among the strongest known selections observed in humans, namely, lactase selection in northern Europeans and sickle-cell trait in Africans. Using inaccurate Amerindian training samples was a major concern for the credibility of previously reported selection signals in Latinos. Taking advantage of the flexibility of our statistical model, we devised a model fitting technique that can learn Amerindian ancestral haplotype from the admixed samples, which allows us to infer local ancestries for Mexicans using only European and African training samples. The strong selection signal at the MHC remains without Amerindian training samples. Finally, we note that medical history studies suggest such a strong selection at MHC is plausible in Mexicans.

Author Summary

Whether or not there exists recent selection since admixture in Latinos has been a subject of debate. To detect selection signal, a method uniquely applicable to recently admixed samples is local ancestry analysis. We infer local ancestry of admixed samples (in our study, Mexicans), and look for regions where the average ancestry of one ancestry component significantly deviates from its genome-wide average. Inferring local ancestry requires training samples that represent the genuine ancestral source populations. One major concern for previously detected selection signals in Latinos via local ancestry analysis is the inaccuracy of Amerindian training samples. This is partly due to large genetic differences among Amerindian tribes and partly due to the difficulty in obtaining Amerindian training samples. We developed a new method which allows us to learn Amerindian ancestral

haplotypes from Mexican cohorts in the absence of Amerindian training samples. Our work demonstrates the existence of recent strong selection at MHC in Mexicans.

Introduction

In 1492 Columbus discovered America. Europeans, led by the Spaniards, and armed with horses, wheels, germs, and steel, rapidly conquered the New World [1], and promptly Africans were brought there as slave labor. During the past 500 or so years, three populations—Amerindians, Europeans, and Africans—have occupied the same space and time, albeit asymmetrically, and were genetically admixing. Twenty generations later, the majority of the people inhabiting Central America, Caribbean Islands, and South America, such as Mexicans, Puerto Ricans, and Columbians have become an admixture of the three continental ancestral populations. These recently admixed populations are of great interest for modern genetic studies [2].

In 2007, Tang and colleagues analyzed a small cohort of Puerto Rican samples and reported three regions that are under strong recent selection [3]. Using their then state-of-the-art local ancestry inference software Saber [4], Tang and colleagues discovered in Puerto Rican samples genomic regions whose mean local ancestries (averaged over individuals) significantly deviated from the genome-wide average—a hallmark of recent selection for admixed samples. Price and colleagues cautioned that the strong selection discovered by Tang and colleagues might be artifacts and they provided three arguments [5]. First, Saber only models linkage disequilibrium (LD), the non-independence of genetic markers in a population, between adjacently markers and thus may produce unreliable local ancestry estimates in regions that harbor long-range LD. It was noted that all three loci under selection that Tang and colleagues reported are within the long-range LD regions. Second, the Amerindian training samples used by Tang and colleagues, which are Maya and Pima samples from human genetic diversity panel (HGDP) [6], is an inaccurate ancestral population for Puerto Ricans, which might produce artifacts in local ancestry inference. Third, Price and colleagues analyzed a larger sample using their software AncestryMap [7] and did not discover the deviation of local ancestry reported by Tang and colleagues.

We would like to make the following comments. First, the AncestryMap uses the so called ancestry informative markers (AIMs) to infer local ancestry; because that AIMs are sparse and that ancestry informative haplotypes may not contain sufficient number of AIMs, the statistical method underlying AncestryMap is evidently under-powered in detecting local ancestry compared to those that attempt to model haplotypes, particularly more recent model-based methods such as HapMix [8] and ELAI [9]. Therefore, negative results from AncestryMap cannot convincingly refute positive findings by Tang and colleagues. Second, the long-range LD, if properly modeled, will benefit the local ancestry inference, because in regions that harbor long-range LD there are more markers *in sync* to define population specific haplotypes. Although Saber [4] has difficulty with long-range LD, more recent model-based methods, such as ELAI [9], can benefit from long-range LD. Third, inaccurate Amerindian training samples is a challenge in studying local ancestry of Latinos. Amerinidan training samples are rarely found in the public domain; the ones that are available, such as Maya and Pima samples from HGDP [6], have small sample sizes and many samples have non-neglegible European ancestries [10].

In this study we analyzed two datasets whose subjects are of Mexican descent, which we obtained from the database of genotype and phenotype (dbGaP). Our primary motivation is to follow up with selection findings in an early study [9], which discovered signatures of recent selection in HapMap3 [11] Mexican samples based on a departure of local ancestry from the global average. Our second motivation is to report a method that can overcome the technical

challenge presented by inaccurate Amerindian training samples when analyzing local ancestry of Latinos. We devised a novel method to infer local ancestry which allows us to discard Amerindian samples and instead learn Amerindian haplotypes from Mexican samples. The strong selection in the MHC region in Mexicans was confirmed in our study.

Results

We applied for access and downloaded two GWAS datasets of Mexican descent from the dbGaP. One is the Viva La Familiar obesity-diabetes familial risk study (henceforth Viva) that contains 815 individuals from 261 families [12]. The other is the Mexican hypertriglyceridemia study (henceforth Lipid) that contains 2229 unrelated individuals [13]. After stringent QC (see Materials and Methods), we applied ELAI to infer local ancestry of each individual. ELAI outperforms other competing state-of-the-art methods in local ancestry inference [9]. It is also convenient to use as it does not require phasing for either training samples or cohort samples, nor does it require recombination map or global admixture proportions as inputs. Public resources, such as HapMap and 1000 Genomes projects, contain high quality European and African haplotypes, but not Amerindian haplotypes; this makes ELAI even more attractive than others in analyzing Mexican samples.

Patterns of global and local ancestry

In VIVA the global ancestry proportions (that is, the admixture proportions) for Amerindian, European, and African components are 0.484,0.452, and 0.064 respectively. In Lipid the numbers are 0.552,0.409, and 0.039. Compared to Viva, Lipid has a higher Amerindian ancestry proportion and lower European and African ancestry proportions. The sampling location is likely to account for the difference: participants in Lipid were recruited in Mexico City, Mexico, whereas participants in Viva were recruited in Houston, Texas. For each ancestry component, there are substantial variations among individuals (see two triangular plots in Fig 1). For both datasets, the topological resemblance between the triangular plot and the principal component (PC) plot is remarkable. The relative positions of the Mexican outlier individuals are well matched, and an African American individual accidentally recruited in Viva is rather obvious. This suggests that ELAI estimates are sensible, and that using PC to derive admixture proportions has some merits [14]. It is believed that using East Asians as additional proxy to Amerindian training samples may improve the local ancestry inference of Latinos, because Amerindians are genetically more similar to East Asians. Our experience suggests, however, that this practice has little impact, and the PC plots, in which Chinese separate from Amerindians inconsistently in two datasets, seem to corroborate our experience.

We computed at each marker the average dosages separately for each ancestral component by averaging that component over all individuals. The average ancestry dosages were computed differently for Viva to account for relatedness in the sample (see Materials and Methods). Fig 2 shows variation of African average dosages along each autosome. (S1A Fig has average dosages for all ancestries.) The spikes on chromosome 6 in both datasets are rather striking. For Viva, the sample standard deviation (ssd) of average dosages for Amerindian, European, and African components are 0.046,0.043, and 0.024 respectively. The largest deviations, measured by the ssd of average dosages for each ancestry, are 5.4,4.8, and 9.9. The locus whose African average dosage is 9.9 ssd above the mean is inside the MHC region, and under the normal approximation, a 9.9 ssd corresponds to a p-value of 2×10^{-23}, which surpasses any reasonable significant threshold for a genome-wide analysis (in GWAS such a significant threshold is 5×10^{-8}). The same region inside MHC was again identified as significant in Lipid; the largest deviation of African average dosages is 14.8 ssd above the mean, which

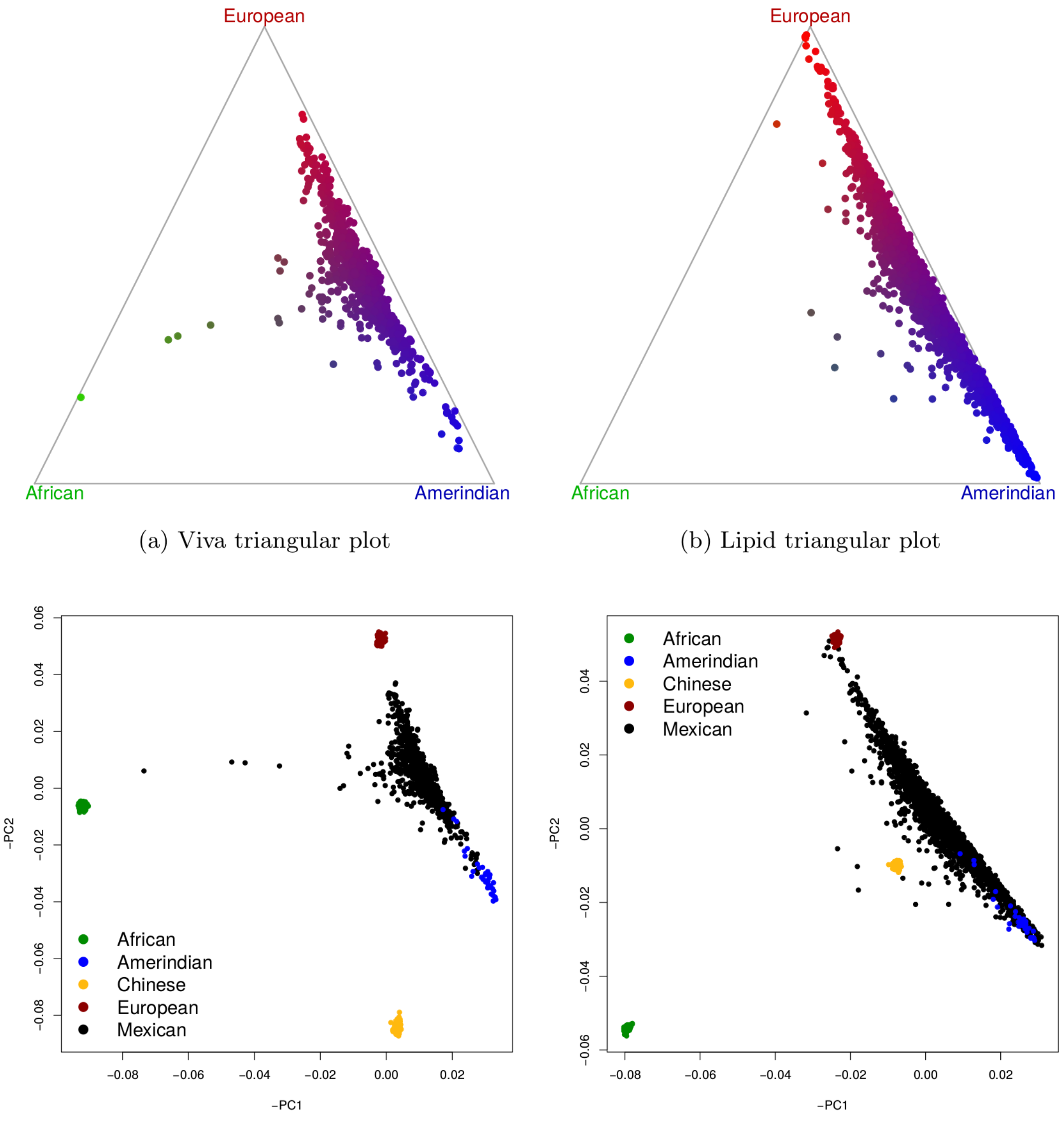

(a) Viva triangular plot

(b) Lipid triangular plot

(c) Viva PC plot

(d) Lipid PC plot

Fig 1. Global ancestry proportions and principal components. (a) and (b) are triangular plots for Viva and Lipid respectively. To produce a triangular plot, note that each individual associates a triplet of ancestry proportions (x, y, z) such that $x + y + z = 1$, and a unique point can be determined such that within an equilateral triangle its distances to three edges are x, y and z. (c) and (d) are PC plots for Viva and Lipid respectively. The PC plots shown are mirror images of the original as indicated by "–" sign in labels.

doi:10.1371/journal.pgen.1005847.g001

corresponds to a p-value of 3×10^{-49}. The region identified in MHC is the same region identified by analyzing HapMap3 Mexican samples [9]. In that study, a region on chromosome 8 was also identified as border-line significant in Amerindian average dosages. In both Viva and Lipid, however, this region was not replicated.

Different European and African training samples and their effects on local ancestry inference

We used HapMap3 Utah Residents with Northern and Western European Ancestry (CEU) as European training samples; Yoruba in Ibadan, Nigeria (YRI), from west Africa, as African

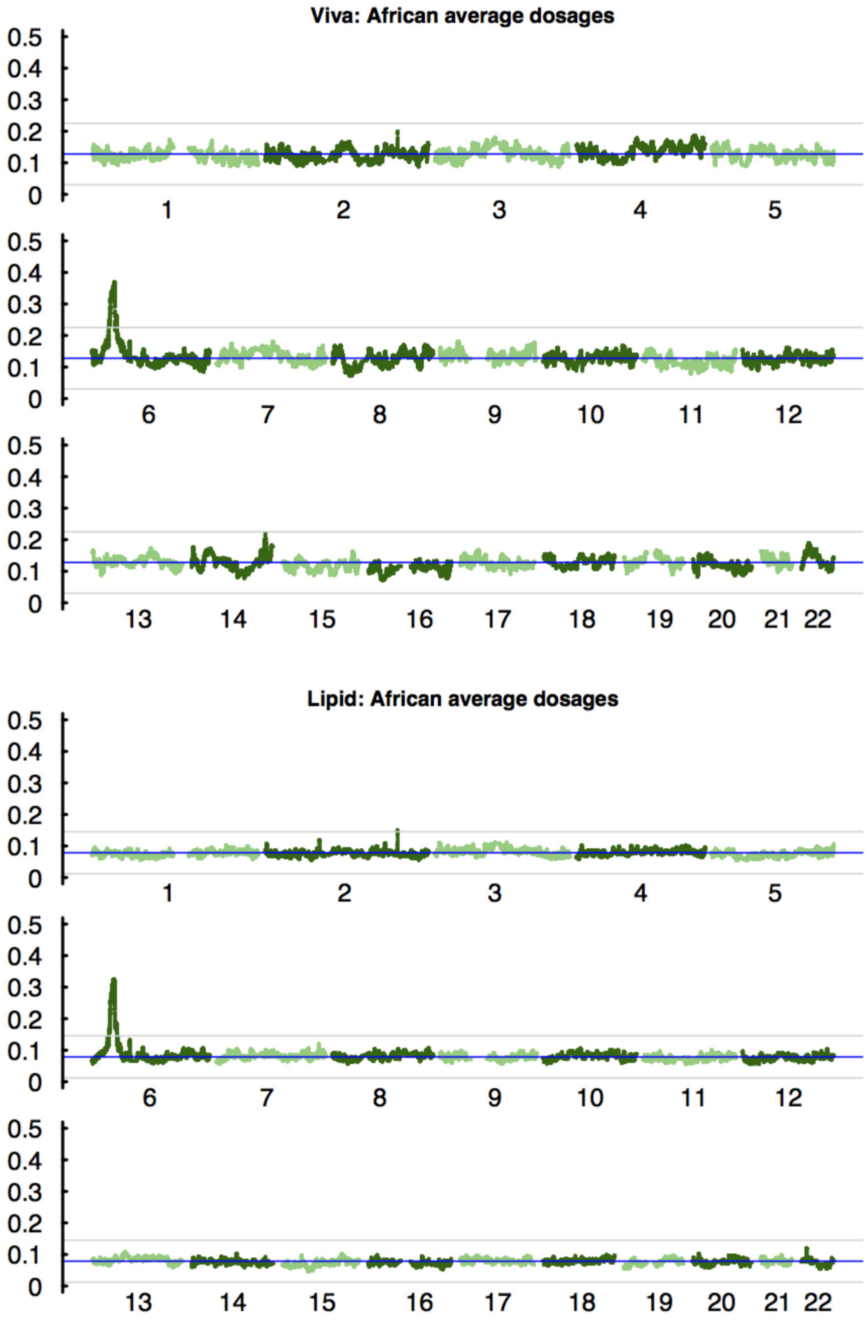

Fig 2. African average dosages. Plot shows all 22 autosomes for two GWAS datasets. The spike at MHC region on chromosome 6 is rather striking in both datasets. The blue lines are the genome-wide mean of average dosages; the gray lines are *mean* ± 4*ssd* (ssd stands for sample standard deviation).

doi:10.1371/journal.pgen.1005847.g002

training samples; and Maya and Pima from HGDP [6] (MAYA) as Amerindian training samples. To test the robustness of our results against different choices of training samples, we first investigated European and African training samples as they both have alternative choices in HapMap3. We used Tuscani in Italia (TSI), from south Europe, as an alternative to CEU, and Maasai in Kinyawa, Kenya (MKK), from east Africa, as an alternative to YRI, and these produced four combinations: CEU–YRI–MAYA, CEU–MKK–MAYA, TSI–YRI–MAYA, and

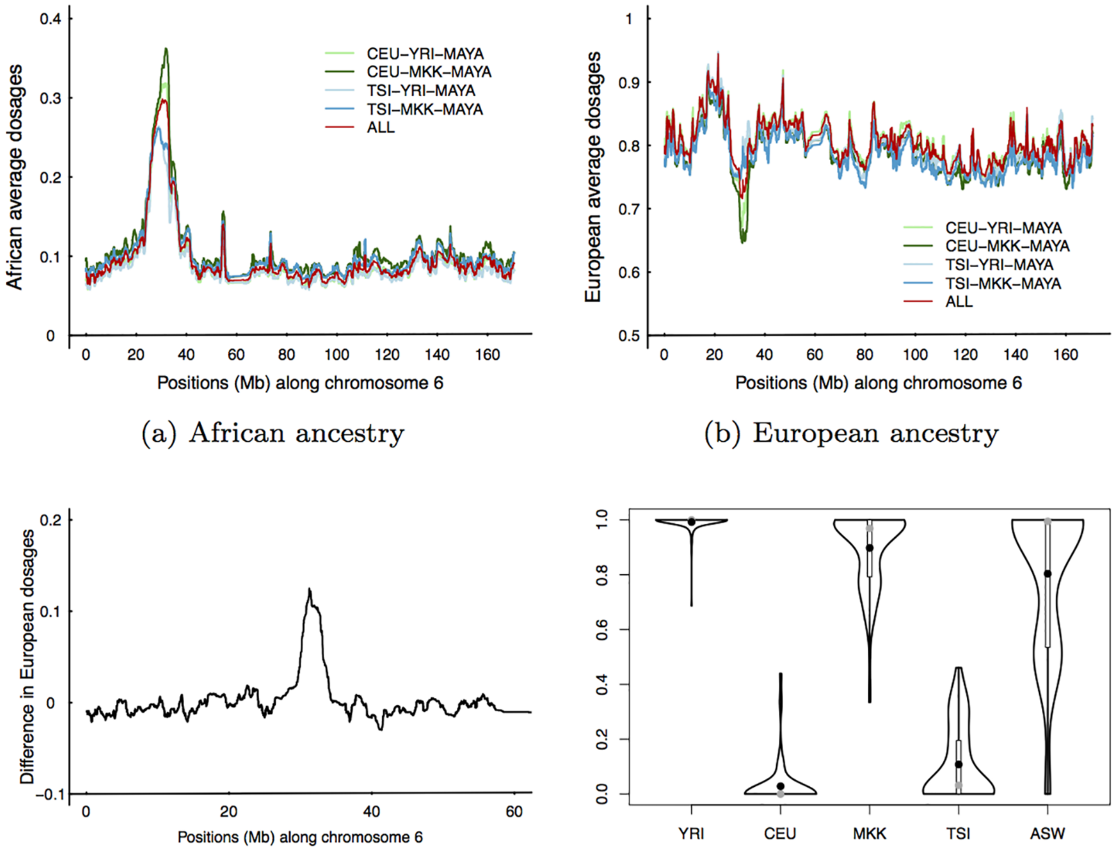

(a) African ancestry

(b) European ancestry

(c) Dosage difference

(d) Population structure

Fig 3. Comparison between different European and African training samples. The comparison was performed with chromosome 6 of Lipid dataset. African (a) and European (b) average dosages for five sets of training samples shown in legend, where ALL means CEU+TSI–YRI+MKK–MAYA. (c) The difference of estimated European average dosages of Mexicans between two European training samples (see main text for explanation). (d) The violin plots of structure analysis of five HapMap3 populations, where ASW denotes Americans from the Southwest, an African American population. On each violin plot, gray dot denotes the median and black dot the mean.

doi:10.1371/journal.pgen.1005847.g003

TSI–MKK–MAYA. We also combined all training samples to perform inference (CEU+TSI –YRI+MKK–MAYA). The genome-wide pattern of local ancestry is consistent for different sets of training samples (S1 Table and S1B and S1C Fig). We thus focus on the MHC region shown in Fig 3(a) and 3(b). We made the following observations: 1) Using TSI to replace CEU produced a less significant deviation at the MHC region. 2) Using MKK to replace YRI produced a more significant deviation at MHC. 3) Combining all training samples produced a significant deviation at MHC, and the significant level is intermediate among other combinations. 4) Outside the MHC region, different combinations of training samples produced congruent results.

Fig 3(c) shows the difference in inferred European average dosages between two European training samples (average difference between TSI–YRI–MAYA vs CEU–YRI–MAYA and TSI-MKK-MAYA vs CEU-MKK-MAYA). Interestingly, the highest peak contains HLA-B and HLA-C loci. We naturally suspect that TSI has more genetic diversity than CEU at the MHC, because more genetically diverse European training samples tend to produce higher estimates of European ancestry dosages. Amerindian average dosages are congruent between choices of CEU and TSI training samples (S2 Fig), and the deficiency in African average dosages when

using TSI as training samples are compensated for by sufficiency of European average dosages. We extracted 8679 SNPs in the extended MHC region, 25–35Mb on chromosome 6, from European and African training samples, and ran ELAI using two upper clusters without specifying the population label, which is essentially haplotype-based structure analysis [9]. One admixture component was arbitrarily chosen to make comparison, and the admixture component was averaged over 10 EM runs (after adjusting for label-switching across EM runs). The violin plots in Fig 3(d) show that TSI is indeed more diverse than CEU at the MHC, MKK is more diverse than YRI, and MKK is the most diverse among four non-admixed populations, which agrees with the theory of east African origin of modern humans [15]. Recently admixed African Americans (ASW) were included for sanity check of the haplotype-based structure inference.

Amerindian training samples and how its removal affects local ancestry inference

Next we turn to Amerindian training samples. The 1000 Genomes admixture analysis group used a collection of Amerindian samples [16] different from the Maya and Pima from HGDP that we used, but we had difficulty in obtaining that data. Moreover, a practical concern is that any specific choice of Amerindian training samples will be subject to suspicion of inaccuracy. To test the robustness of our inference against different Amerindian training samples, we elected to remove Amerindian training samples and used only European and African training samples to perform inference—but of course we kept the setting of three ancestral populations. ELAI can function with the absence of one training population as long as there are enough genetic components of that ancestry in the cohort samples. Because Mexicans have a large Amerindian ancestry proportion, when Amerindian training samples are missing, ELAI is still able to learn Amerindian ancestral haplotypes relatively easily from Mexican samples as long as the sample size is large. The same is true for European training samples, but it becomes more difficult if African training samples are missing. To borrow an analogy from next-generation sequencing, a large number of Mexican samples and a high ancestry proportion to local ancestry inference is analogous to a high coverage of sequencing reads to variant call.

The recommended practice in an early version of ELAI is to split a large dataset into small subsets. Doing so not only improves computational efficiency on a computer cluster, but also allows ELAI to jointly fit training and cohort datasets. It is evident [17, 18] that a cluster model becomes less fit to the training samples in the presence of an overwhelmingly large number of cohort samples, which undermines the performance of local ancestry inference (or imputation). Recall that removing Amerindian training samples requires a large number of cohort samples jointly fitting the model with training samples—we are seemingly in a quandary. The solution is rather simple. In parameter estimation of the two-layer model underlying ELAI [9], we can arbitrarily adjust relative weights between cohort and training samples without changing the expected ancestral allele (haplotype) frequency estimates. In other words, we can take an arbitrarily large number of cohort samples and down weight their contribution to parameter estimation. When the training samples are available, the weighting ensures the model fits to training samples sufficiently; otherwise, the ancestral alleles are estimated exclusively by cohort samples, and the weight cancels out in the parameter estimation as long as we assign equal weight to all cohort samples. (The technical details can be found in Materials and Methods.) Thus, the weighting allows us to take the extreme measure of removing Amerindian training samples.

We implemented the weighting scheme and applied it to both datasets. We combined CEU and TSI as European training samples and YRI and MKK as African training samples. Fig 4

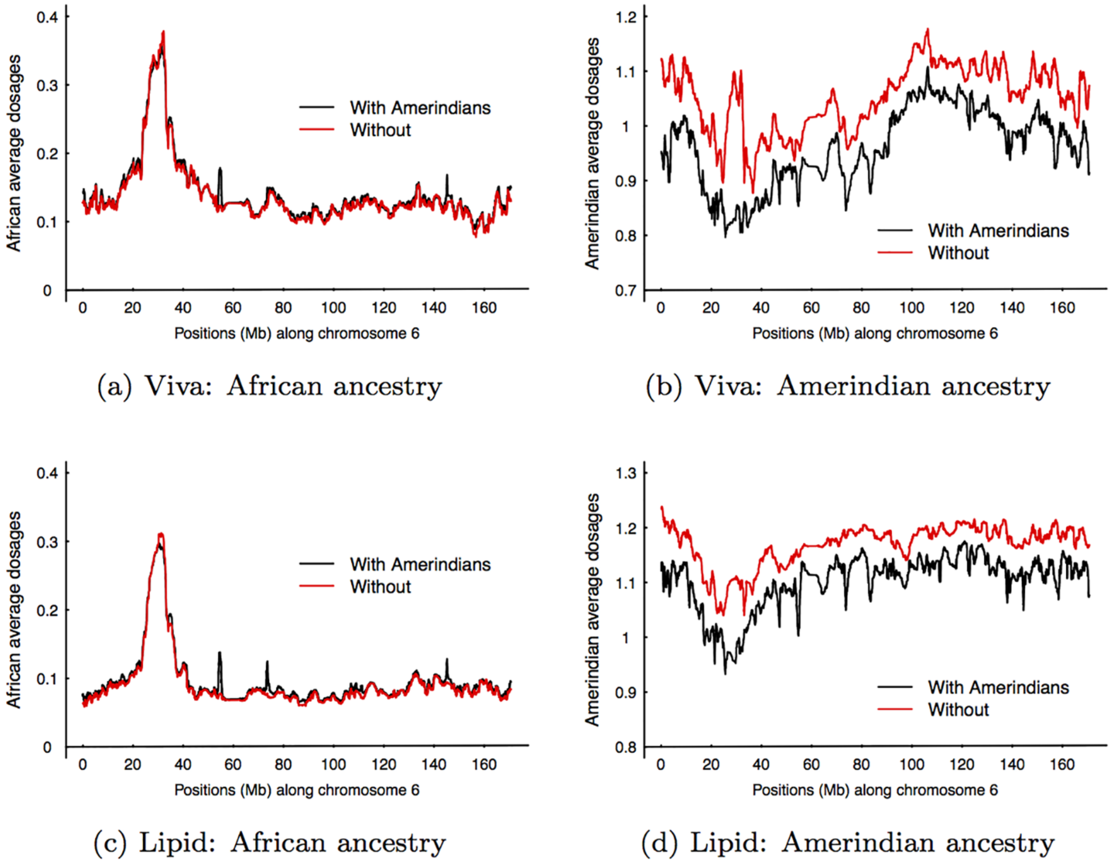

Fig 4. Comparison of estimations with and without Amerindian training samples. (a) African average dosages of Viva. (b) Amerindian average dosages of Viva. (c) African average dosages of Lipid. (d) Amerindian dosages of Lipid. We combined CEU and TSI as European training samples, and YRI and MKK as African training samples.

doi:10.1371/journal.pgen.1005847.g004

demonstrates, using both Viva and Lipid datasets, the difference, or lack of it, in the estimated African average dosages with and without Amerindian training samples. Comparing the Amerindian average dosages, however, the estimates without Amerindian training samples are higher than that with. The mean differences are 0.09 for Viva and 0.08 for Lipid. This is not too surprising considering 1) Maya and Pima samples have some European ancestral components (PC plots in Fig 1); and 2) Maya and Pima samples may be imperfect representatives of the Amerindian source populations for Mexicans, and learning Amerindian ancestry components from a large number of cohort samples may provide a better fit. Our results shall eliminate concerns of possible artifacts caused by inaccurate Amerindian training samples.

Strong selection at the MHC region

If purely by chance, it is very unlikely that Amerindians share more alleles with Africans at MHC than the rest of the genome at such a significant level; that the pathogens from the Old world are often lethal to the native inhabitants of the New World seems to argue against such a peculiar sharing. The effect of the population bottleneck and the drift do not distinguish the MHC from the rest of the genome [19]. If selection happened in Africans before admixture, one would expect to see such selection signals in African Americans, which are not there [20]. Therefore, it is safe to assume that the African average dosages in Mexicans rose from the genome-wide mean p_0, which is a proxy dosage before selection at MHC, to the inferred value

Table 1. Estimates of selection coefficient *s* under different models. p_0 is the genome-wide mean of African average dosages; p_1 is the peak African average dosage at MHC.

Data	p_0	p_1	*s*		
			Simple	Dominance	Additive
Viva	0.128	0.365	0.054	0.069	0.061
Lipid	0.079	0.325	0.073	0.089	0.081

doi:10.1371/journal.pgen.1005847.t001

of p_1 at MHC in the past 20 generations, and it is selection at work. A selection coefficient *s* can be computed via a simple model $p_1 = p_0 \times (1+s)^{20}$, which provides a lowerbound estimate of *s* compared to recursion formula for both dominance and additive models (see Materials and Methods). Table 1 summarizes the estimates of selection coefficient under different models; the lower-bound estimates are $s = 0.05$ for Viva and $s = 0.07$ for Lipid. Both estimates indicate a very strong selection, on par with the lactase selection in northern Europeans (0.09–0.19) [21] and the sickle-cell trait in Africans (0.05–0.18) [22].

To understand how many SNPs have contributed to the selection signal in MHC, we assigned a phenotypic value to each individual based on their African ancestry dosage at the identified region in MHC (detailed in Materials and Methods), regressed out six leading principal component and admixture proportions, and performed the single-SNP association test using BIMBAM [17]. At a very liberal threshold of $\log_{10}$ Bayesfactor > 10, we discovered 1700 SNPs in the extended MHC region to be genome-wide significant (S3 Fig). Considering the high correlation among SNPs in the region, we next performed multi-SNP analysis using a Bayesian variable selection regression procedure implemented in the software piMASS [23]. piMASS implements a Markov chain Monte Carlo (MCMC) procedure to sample the posterior distribution of model space (SNP sets) under sparse and shrinkage priors. The output contains posterior probability of association (PPA) for each SNP, which roughly reflects how often the SNP is being selected in an additive model. We ran piMASS using all markers from chromosome 6 of Lipid with 10,000 burn-in steps and 1 million sampling steps. Two independent runs were conducted. In both runs, the proportion of variation explained (the narrow sense heritability) estimates had the same posterior mean of 0.88, with ssd of 0.015 and 0.017 respectively. The posterior mean model sizes (the number of SNPs in the model sampled) were 93±10.7 and 83±7.1 respectively (mean ± ssd). The two runs had 126 and 116 SNPs with PPA >0.1; among them, 60 SNPs overlapped, and the union contained 182 SNPs. We removed these 182 SNPs and reran local ancestry inference of chromosome 6. The pattern of the local ancestry was essentially unaffected. These exercises suggest that the observed selection signal is driven by a large number of SNPs and their constitutional haplotypes.

Discussion

In this paper we analyzed two existing GWAS datasets of Mexican subjects and demonstrated that the MHC region is under strong recent selection in Mexicans. Because Viva contains related individuals, we split individuals into non-overlapping subsets, each containing 40–50 unrelated individuals; performed local ancestry inference separately for each subset; and aggregated them to compute the average dosages. This practice produced congruent results as our combined analysis. In Lipid, samples were assigned case-control labels according to their triglyceride levels. The results presented in the paper ignored the case-control status. We analyzed cases and controls separately, and the results were highly congruent to that of the combined analysis. We also analyzed African American samples in HapMap3 and did not find any region under selection, which agrees with a recent study [20]. This serves as a negative control for

ELAI. We devised a model fitting technique to introduce weighting into parameter estimation, which makes it possible to infer local ancestry of Mexicans using only European and African training samples. This rids us of the concern that the detected selection signals in Mexicans are artifacts produced by inaccurate Amerindian training samples.

A previous study detected selection in 1000 genomes Mexican samples through local ancestry analysis [9]. Bhatia and colleagues questioned the plausibility of that finding; they argued that if signals were there, the 1000 genomes admixture analysis group would have found it [20]. We took this opportunity to investigate why the 1000 genomes admixture analysis group failed to detect the strong selection at the MHC region in Mexicans. We simulated genotypes using a demographic model that mimic the out-of-Africa migration events [24], performed forward simulations to mimic admixture and selection at three linked loci (details in Materials and Methods), and inferred local ancestry. The 1000 genomes used consensus call from four programs: HapMix [8], LAMP-LD [25], RFMix [26], and MultiMix [27]. The publicly available version of HapMix was designed exclusively for two-way admixture, and the extended version used to analyze the 1000 Genomes data was not available to us [28]. Thus it was excluded from our analysis. MultiMix performed poorly despite our best effort and was excluded as well. For both LAMP-LD and RFMix we used the same parameter settings as those used in the 1000 Genomes admixture analysis group [28]. Both LAMP-LD and RFMix require phased training samples, and RFMix also requires phased cohort samples. (ELAI works with diplotypes.) When supplied with true phasing, both LAMP-LD and RFMix works well, on par with ELAI. We then introduced 2% switch-errors into cohort haplotypes and training haplotypes that mimic Amerindians, 1% switch-errors into European and African training samples. LAMP-LD is robust to switch-errors, but RFMix under-performs (S4A and S4B Fig). It is worthwhile to note that MHC is notoriously hard to phase, and phasing for admixed samples at MHC is even more challenging as it requires the phasing algorithm to correctly identify local ancestry—a catch-22 for RFMix. We were surprised at the worse-than-the-expected performance of RFMix in the presence of switch-errors (S4C Fig). Further investigation revealed that its window size parameter has a sweet-spot (S4D Fig). When using the best window size RFMix performed on par with ELAI (S4E Fig). Going back to the question why the 1000 genomes admixture analysis group failed to detect the signal, our simulation studies suggested that the democratic strategy adopted by 1000 genomes admixture analysis group, which used consensus calls from four methods to identify local ancestry, was perhaps not optimal. The simulation studies prompted us to use LAMP-LD and RFMix to analyze chromosome 6 of Viva and Lipid data. We phased the Maya and Pima samples from HGDP using SHAPEIT [29], which were used in combination with CEU and YRI haplotypes as training datasets. LAMP-LD was then applied to infer local ancestry of Viva and Lipid datasets. We then phased the Viva and Lipid datasets, and RFMix was applied to infer their local ancestry. Reassuringly, both LAMP-LD and RFMix discovered the signal of selection at MHC (S5 Fig).

The MHC region influences susceptibility and resistance to a broad range of infectious agents such as viruses, bacteria, and parasites. It is sensible to observe more alleles of African ancestry at MHC in Mexicans if those alleles confer selective advantages in the presence of certain infectious agents. The European conquerors brought to America European and African diseases such as smallpox, measles, and typhus. Spaniards imposed an urbanized life style and farming practice on native people. A sudden increase in local population concentration, displacement, social upheaval, food shortages, and stress made them much vulnerable to infectious diseases. An estimated 5–8 million native people perished in a smallpox epidemic alone in early 1500s [30]. Nevertheless, after "difficult struggles of the formative period," the acceptance and enthusiasm of the new life emerged from the persistence of the old; for a brief period a "fusion of European and Mesoamerican cultures seemed ready to emerge" [31]. But severe

drought hit and lethal pandemic broke out [30, 31]. The epidemic, called "huey cocoliztli," was symptomatically different from those imported from the Old World; some medical historians suspect it was a hemorrhagic fever caused by arenavirus carried by rodents [31]. It first broke out in 1545 and lingered until 1815 [31, 32]. The epidemic selectively targeted native people, and 90% of the population perished in a few generations [30, 32]. This sustained epidemic harbors plenty of opportunities for strong selection at MHC, which fits our analysis. Once again history left its mark in genomes for posterity [33].

Materials and Methods

Datasets

The first dataset, Viva La Familia obesity-diabetes familial risk study (dbGaP Study Accession: phs000616.v1.p1), contains 858 genotyped individuals [12]. Among them, 815 Mexicans children from 261 families were genotyped with Illumina HumanOmni 1-v1.0 BeadChips, and the remaining 43 children were genotyped on HumanOmni 2.5–8v1 BeadChips. We chose to analyze the 815 samples that were typed on the same chip. Study participants in Viva La Familiar study were recruited in Houston, Texas. The second dataset, Mexican hypertriglyceridemia study (dbGaP Study Accession: phs000618.v1.p1), contains 2229 samples with 1117 cases and 1112 controls, where the case–control status was ascertained based on an individual's serum triglyceride level [13]. Note that although there were 4350 study samples reported in the paper, the dbGaP contains only 2229 that were genotyped with Illumina Human610-Quad BeadChips—stage 1 of the GWAS. The rest samples were only typed on selected 1200 SNPs—stage 2. Study participants in this study were recruited in Mexico City. We call the first dataset Viva and the second Lipid.

Data quality control

We removed all A/T, C/G SNPs whose potential allele flipping between different datasets cannot be identified without additional information. A SNP was removed if it was missing in one of the datasets, either training or cohort. We also removed SNPs whose missing proportion was larger than 5%. Although we realized that the Hardy-Weinburg disequilibrium test is not appropriate for admixed samples, we used it anyway to remove SNPs whose HWD test p-values $<10^{-6}$. It is understood that this practice errs toward the safe side by eliminating possibly good SNPs. Finally, we obtained the cluster plots for each SNP, devised a simple algorithm to assign quality scores to each SNP cluster plot, and visually inspected those SNPs whose score indicated low quality. We removed those SNPs that contained a fourth cluster, or whose clusters were not distinct (examples of such cluster plots can be found in [34]). We were particularly stringent to conduct such SNP quality control at the MHC region. Of the two GWAS datasets we obtained from dbGaP, Viva contains SNP cluster information, but Lipid does not. In the end, we had 352,754 SNPs from Viva and 479,757 SNPs from Lipid. The low number of SNPs in Viva reflected small number of overlapping SNPs between the Illumina HumanOmni 1-v1.0 and the Illumina 650Y arrays, the latter of which was used by the HGDP study that generates the Maya and Pima genotypes used as Amerindian training samples.

Local ancestry inference

We used ELAI [9] for local ancestry inference, which has been demonstrated to outperform competing methods such as HapMix [8] and LAMP-LD [25]. ELAI implements a two-layer cluster model and the model is fitted via the EM algorithm. The upper-layer clusters are parameterized to represent haplotypes from ancestral populations, and the lower-layer clusters

contemporary haplotypes. The two-layer model was motivated by approximating the coalescent with recombination. It directly applies to diplotypes and automatically integrates out phase uncertainty. It can also estimate the recombination rates between markers, and hence doesn't require recombination map as an input. Thus, the requirement for running ELAI is minimal—just genotypes and marker positions. To run ELAI, one needs to provide training samples. We used European and African samples from HapMap3 and Maya and Pima samples from HGDP as default training samples (or reference panels, or source populations). ELAI is a cluster-based model and we wanted to specify numbers of clusters. The number of upper-layer clusters represents the number of source populations and we set it as 3; the number of lower-layer clusters was set as 15. Extensive simulations demonstrated that this parameter setting performs well [9]. Lastly, we needed to specify number of admixing generations and we used 20. All ELAI results were averaged over 10 independent EM runs of 20 steps each, unless noted.

Compute average ancestry dosages

Lipid data contains unrelated individuals, and we treated an individual as unit and the computation is straightforward. Viva data contains 261 unrelated families. Each family contains 1–8 children, with majority of families (242) having 2–4 children. To account for relatedness in Viva data, we treat a family instead of an individual as unit, and computed the average dosages in the following manner: first we obtained family ancestral dosages by averaging over family members, and then we averaged over families to obtain overall average dosages.

Assign different weights to training and cohort samples

The two-layer model and the details of model fitting using EM algorithm can be found in [9]. Here we show how to estimate θ, the allele frequency associated with the cluster which emits the observed data. To simplify notation and presentation, we assume observing haplotypes instead of diplotypes. The weighting scheme can be applied to mixed sample that contains both haplotypes and diplotypes. To update parameters in each EM step, we take derivative of the expected full data log likelihood with respect to a parameter we want to update, say $x \in \xi$,

$$\frac{d}{dx} E_{Z^{(1)},\ldots,Z^{(n)}|h^{(1)},\ldots,h^{(n)},\xi^*} \left[\log p(h^{(1)}, \ldots, h^{(n)}, Z^{(1)}, \ldots, Z^{(n)}|\xi) \right] = 0, \tag{1}$$

and solve for x to obtain updates. $Z^{(i)}$ is the latent state of haplotype $h^{(i)}$, which contains two components, one for each layer of clusters. The expectation in Eq (1) is with respect to the posterior probability of latent states, conditioning on ξ^*, which is the collection of parameters of the two-layer model estimated from the previous iteration, and ξ is the collection of parameters to be estimated. At marker m, write $q_{ij} = \sum_s p(Z_m = (s, j)|h_m^{(i)}, \xi^*)$, which is the marginal posterior probability of $h_m^{(i)}$ emitted from cluster j. Let $T_k = \{i : h_m^{(i)} = k\}$ for $k = 0,1$. Take the derivative with respect to θ_{mj}, which is the allele frequency associated with cluster j, to get

$$\frac{-1}{1 - t_j} \sum_{i \in T_0} q_{ij} + \frac{1}{t_j} \sum_{i \in T_1} q_{ij} = 0, \tag{2}$$

and solve to get

$$t_j = \frac{\sum_{i \in T_1} q_{ij}}{\sum_{i \in T_0} q_{ij} + \sum_{i \in T_1} q_{ij}}, \tag{3}$$

which can be thought as estimates of θ_{mj} with *equal* weight 1. To apply differential weights, we split T_k into training sample $T_k^{(t)}$ and cohort sample $T_k^{(c)}$. For training sample we assign a weight

w_t and for cohort sample w_c. Eq (3) is generalized to

$$t_j = \frac{w_t \sum_{i \in T_1^t} q_{ij} + w_c \sum_{i \in T_1^c} q_{ij}}{w_t \left(\sum_{i \in T_0^t} q_{ij} + \sum_{i \in T_1^t} q_{ij} \right) + w_c \left(\sum_{i \in T_0^c} q_{ij} + \sum_{i \in T_1^c} q_{ij} \right)}. \quad (4)$$

Let $w_t \gg w_c$, then cohort samples contribute very little to t_j when training samples are present. This is often desirable because the q_{ij} estimates of training samples are more reliable, which is especially true in the context of imputation [17]. When training samples are missing, the first terms of both nominator and denominator on the right hand side which involve w_t disappear and Eq (4) reduces to Eq (3).

Using simulated data (described below), we fit the ELAI model using two training samples of European and African, discarding the Amerindian training samples. The African ancestral dosages were used to compare the inferred values and the truth. The results demonstrated that the weighting samples works well for selection coefficients of 0.02 and 0.05, and showed a bias for selection coefficient of 0.10, but the biased estimates were conservative for the purpose of detecting selection (S6 Fig).

Define phenotype for association test

We defined a marker set A that contained markers whose African average dosages were greater than 0.30. This threshold was 13 sample standard deviations away from the mean (in Lipid dataset), and the resulting markers formed a consecutive region within MHC. We assigned each individual a phenotypic value obtained by averaging African ancestry dosages over markers in A.

Compute selection coefficient

Let s be the selection coefficient, and $f_n(s)$ denote allele frequency at the n-th generation which is a function of s. Here the allele is referred to as a class of population specific alleles. Assume that the population size is constant but infinite so that we have a deterministic model. For dominance model where both heterozygous individual and homozygous individual of advantageous alleles has the same fitness 1+s, we have recursion $f_{n+1}(s) = \frac{f_n(s)(1+s)}{1+(2-f_n(s))f_n(s)\ s}$. For additive model where a heterozygous individual has fitness 1+s and a homozygous individual of advantageous alleles has fitness 1+2s, we have recursion $f_{n+1}(s) = \frac{f_n(s)(1+s+f_n(s)\ s)}{1+2f_n(s)\ s}$. Let $n = 20$; we know the values of $f_0(s)$ and $f_{20}(s)$ and we want to find s. Because $f_n(s)$ is a monotone function of s, we perform interval-bisection search to numerically solve for s. We start with an interval [a, b], such that $f_{20}(a) < f_{20}(s) < f_{20}(b)$, we evaluate $y = f_{20}\left(\frac{a+b}{2}\right)$, if $y > f_{20}(s)$, we set $b = y$; otherwise we set $a = y$. We repeat this procedure until $y - f_{20}(s) \in (-\epsilon, \epsilon)$ for a small ϵ. Note that to apply the recursion formulae, the input $f_0(s)$ and $f_{20}(s)$ have to be allele frequencies, which are half of the allele dosages for humans.

We call the model defined by recursion $f_{n+1}(s) = f_n(s)(1+s)$ the *simple model*. It is easy to check that for dominance model we have $f_{n+1}(s) = \frac{f_n(s)(1+s)}{1+(2-f_n(s))f_n(s)\ s} < f_n(s)(1+s)$; and for additive model we have $f_{n+1}(s) = \frac{f_n(s)(1+s+f_n(s)\ s)}{1+2f_n(s)\ s} < f_n(s)(1+s)$. Therefore the simple model produces a lower-bound estimate of s for both dominance and additive models. Let $f_0(s) = p_0$ and $f_{20}(s) = p_1$, we have $p_1 = p_0(1+s)^{20}$, and therefore the simple model estimate of selection coefficient is $s = \exp(\log(p_1/p_0)/20) - 1$.

Simulate admixed samples under selection

We used a population genetics model that mimics the out-of-Africa migration events to simulate a 3 Mb region of three source populations that mimic Amerindian, European, and African

[24]. After setting aside 200 haplotypes from each source population as training haplotypes, we used the remaining haplotypes to simulate three-way admixed individuals by a one-pulse model [35]. Specifically, we randomly selected 50,000 haplotypes from the three source populations using proportions of 50%, 45%, and 5%, mimicking the admixture proportion of Mexicans. We split 3 Mb into three segments, and assigned at two splitting points recombination hotspots. At each hotspot, we assumed equal recombination probability of 0.1,0.2, and 0.5 per generation. We sampled two haplotypes with replacement and introduced possible crossover events at hotspots to produce two new haplotypes. We repeated the pairing and crossover 25,000 times to produce 50,000 haplotypes for the next generation. The admixture simulation was done for 20 generations. To simulate selection, we designated the mid-section as the locus under selection, and assumed selection coefficients of 0.02,0.05, and 0.10 per generation. The alleles under positive selection were those 5% from the source population that mimicked Africans. After 20 generations, we randomly chose 2,000 admixed haplotypes, pairing them to form 1,000 diplotypes as cohort samples. We used two sizes of mid-section: 0.5 Mb and 1 Mb. A small mid-section produces a more challenging problem. To investigate how switch-errors affected local ancestry inference for different methods, in addition to perfect phasing situation, we also introduce 2% phasing errors into Amerindian training samples and the cohort samples, and 1% phasing errors to European and African training samples. To do so, at randomly selected heterozygous marker, from left to right we crossed-over two haplotypes.

Supporting Information

S1 Table. Summary statistics for different sets of training samples for autosomes of the Lipid dataset. ALL means CEU+TSI−YRI+MKK−MAYA, and ssd means sample standard deviation.
(PDF)

S1 Fig. Average dosages with different European and African training samples. A) Average dosages for Amerindian (blue), European (red), and African (green) ancestries for Viva (top) and Lipid (bottom) datasets with training samples of CEU−YRI−MAYA. B) Average dosages for Amerindian (blue), European (red), and African (green) ancestries for Lipid dataset with training samples of CEU−MKK−MAYA (top) and TSI−MKK−MAYA (bottom). C)Average dosages for Amerindian (blue), European (red), and African (green) ancestries for Lipid dataset with training samples of TSI−YRI−MAYA (top) and CEU+TSI−YRI+MKK−MAYA (bottom).
(PDF)

S2 Fig. Comparison of Amerindian average dosages. The five sets of training samples are shown in the legend, where ALL means CEU+TSI−YRI+MKK−MAYA. The comparison was performed with chromosome 6 of Lipid dataset.
(PDF)

S3 Fig. SNPs associated with enrichment of African local ancestry in Lipid dataset. Bayes factors (BF) were computed using BIMBAM. The horizontal blue line is $\log_{10} BF = 10$.
(PDF)

S4 Fig. Simulation studies to evaluate LAMP-LD and RFMix. A) Comparison between LAMP-LD, RFMix, and ELAI under different simulation conditions, Part I. There are 9 combinations of crossover probability (0.1,0.2, and 0.5) and selection coefficients (0.02,0.05, and 0.10) for two sizes of the mid-section. This plot is for mid-section of size 1 Mb. The mid-section harbors alleles under selection, and a smaller size produces a more challenging problem. Plots also compare effects of phasing errors (2% for cohort and the Amerindian training sample and

1% for the other two training samples). RFMix underperformed after phasing errors were introduced in (b). Compared to RFMix, LAMP-LD was less sensitive to phasing errors. ELAI was unaffected by phasing errors. Parameters for LAMP-LD: window size = 100, number of HMM states = 25; for RFMix: window size = 0.1 cM, which approximately contains 100 SNPs. Both parameter settings were used by the 1000 Genomes admixture analysis group. **B)** Comparison between LAMP-LD, RFMix, and ELAI under different simulation conditions, Part II. The same simulation setup as in S4A Fig but with mid-section of size 0.5 Mb. **C)** RFMix performance with different switch-errors. This is the same dataset as used in S4B Fig. In the legends, the number before the plus sign is the switch-error for cohort and the Amerindian training sample, and the number after is for the European and African training samples. **D)** RFMix performance with different choices of window size. The mean absolute deviation was computed from the same dataset that was used in S4B Fig and averaged over 9 simulation parameter settings (recombination probability and selection strength). The switch-errors were 2% for cohort and the Amerindian training sample and 1% for the other two training samples. We used centi-Morgan (cM) to measure the window size, which is the unit used by RFMix. In our simulations, 1 cM contains roughly 1000 SNPs. **E)** RFMix performs well with the optimal window size. This is the same dataset as used in S4B Fig. When the optimal window size is used, RFMix performs well in the presence of phasing errors (2% for cohort and the Amerindian training sample and 1% for the other two training samples).
(PDF)

S5 Fig. Average dosages inferred by LAMP-LD and RFMix of chromosome 6 of Viva and Lipid datasets. LAMP-LD (first column) and RFMix (second column) discovered excessive African average dosages at MHC for both Viva (first row) and Lipid (second row) datasets.
(PDF)

S6 Fig. Simulation studies to exclude Amerindian training samples. The grey lines indicate the truth. The black lines are the inferred African average dosages by ELAI with only European and African training samples. There are 9 combinations of crossover probability (0.1,0.2, and 0.5) and selection coefficients (0.02,0.05, and 0.10), and the size of the mid-section is 0.5 Mb. On each plot the main text displays the simulation parameters with C for crossover probability and S for selection coefficient. For example, $C = 0.2$, $S = 0.05$ means crossover probability is 0.2 and selection coefficient is 0.05.
(PDF)

Acknowledgments

We thank editors and two anonymous referees for comments that greatly improved the presentation. Mark Meyer provided thorough editorial assistance of the manuscript.

Author Contributions

Conceived and designed the experiments: YG. Performed the experiments: LZ QZ. Analyzed the data: QZ LZ YG. Contributed reagents/materials/analysis tools: YG. Wrote the paper: YG. Weighting scheme in ELAI used in analysis: YG. Forward simulation program used in analysis: LZ.

References

1. Diamond J. Guns, Germs, and Steel: The Fates of Human Societies. W.W. Norton and Company; 1997.

2. González Burchard E, Borrell LN, Choudhry S, Naqvi M, Tsai HJ, Rodriguez-Santana JR, et al. Latino Populations: A Unique Opportunity for the Study of Race, Genetics, and Social Environment in Epidemiological Research. American Journal of Public Health. 2005 12; 95(12):2161–2168. Available from: http://www.ncbi.nlm.nih.gov/pmc/articles/PMC1449501/. doi: 10.2105/AJPH.2005.068668 PMID: 16257940

3. Tang H, Choudhry S, Mei R, Morgan M, Rodriguez-Cintron W, Burchard EG, et al. Recent Genetic Selection in the Ancestral Admixture of Puerto Ricans. American Journal of Human Genetics. 2007 09; 81(3):626–633. Available from: http://www.ncbi.nlm.nih.gov/pmc/articles/PMC1950843/. doi: 10.1086/520769 PMID: 17701908

4. Tang H, Coram M, Wang P, Zhu X, Risch N. Reconstructing Genetic Ancestry Blocks in Admixed Individuals. American Journal of Human Genetics. 2006 07; 79(1):1–12. Available from: http://www.ncbi.nlm.nih.gov/pmc/articles/PMC1474129/. doi: 10.1086/504302 PMID: 16773560

5. Price AL, Weale ME, Patterson N, Myers SR, Need AC, Shianna KV, et al. Long-Range LD Can Confound Genome Scans in Admixed Populations. American Journal of Human Genetics. 2008 07; 83(1):132–135. Available from: http://www.ncbi.nlm.nih.gov/pmc/articles/PMC2443852/. doi: 10.1016/j.ajhg.2008.06.005 PMID: 18606306

6. Li JZ, Absher DM, Tang H, Southwick AM, Casto AM, Ramachandran S, et al. Worldwide Human Relationships Inferred from Genome-Wide Patterns of Variation. Science. 2008; 319(5866):1100–4. doi: 10.1126/science.1153717 PMID: 18292342

7. Patterson N, Hattangadi N, Lane B, Lohmueller KE, Hafler DA, Oksenberg JR, et al. Methods for High-Density Admixture Mapping of Disease Genes. American journal of human genetics. 2004 05; 74(5):979–1000. Available from: http://linkinghub.elsevier.com/retrieve/pii/S0002929707643638. doi: 10.1086/420871 PMID: 15088269

8. Price AL, Tandon A, Patterson N, Barnes KC, Rafaels N, Ruczinski I, et al. Sensitive Detection of Chromosomal Segments of Distinct Ancestry in Admixed Populations. PLoS Genet. 2009 06; 5(6):e1000519. doi: 10.1371/journal.pgen.1000519 PMID: 19543370

9. Guan Y. Detecting Structure of Haplotypes and Local Ancestry. Genetics. 2014; 196(3):625–642. Available from: http://www.genetics.org/content/196/3/625.abstract. doi: 10.1534/genetics.113.160697 PMID: 24388880

10. Johnson NA, Coram MA, Shriver MD, Romieu I, Barsh GS, London SJ, et al. Ancestral Components of Admixed Genomes in a Mexican Cohort. PLoS Genet. 2011 12; 7(12):e1002410. doi: 10.1371/journal.pgen.1002410

11. The International HapMap Consortium. Integrating common and rare genetic variation in diverse human populations. Nature. 2010 09; 467(7311):52–58. doi: 10.1038/nature09298 PMID: 20811451

12. Comuzzie AG, Cole SA, Laston SL, Voruganti VS, Haack K, Gibbs RA, et al. Novel Genetic Loci Identified for the Pathophysiology of Childhood Obesity in the Hispanic Population. PLoS ONE. 2012 12; 7(12):e51954. doi: 10.1371/journal.pone.0051954 PMID: 23251661

13. Weissglas-Volkov D, Aguilar-Salinas CA, Nikkola E, Deere KA, Cruz-Bautista I, Arellano-Campos O, et al. Genomic study in Mexicans identifies a new locus for triglycerides and refines European lipid loci. Journal of Medical Genetics. 2013; 50(5):298–308. Available from: http://jmg.bmj.com/content/50/5/298.abstract. doi: 10.1136/jmedgenet-2012-101461 PMID: 23505323

14. Brisbin A, Bryc K, Byrnes J, Zakharia F, Omberg L, Degenhardt J, et al. PCAdmix: Principal Components-Based Assignment of Ancestry along Each Chromosome in Individuals with Admixed Ancestry from Two or More Populations. Hum Biol. 2012 Aug; 84(4):343–364. Available from: http://www.ncbi.nlm.nih.gov/pmc/articles/PMC3740525/. doi: 10.3378/027.084.0401 PMID: 23249312

15. Cavalli-Sforza LL, Feldman MW. The application of molecular genetic approaches to the study of human evolution. Nat Genet. 2003; 33:266–275. doi: 10.1038/ng1113 PMID: 12610536

16. Mao X, Bigham AW, Mei R, Gutierrez G, Weiss KM, Brutsaert TD, et al. A Genomewide Admixture Mapping Panel for Hispanic/Latino Populations. American Journal of Human Genetics. 2007 06; 80(6):1171–1178. Available from: http://www.ncbi.nlm.nih.gov/pmc/articles/PMC1867104/. doi: 10.1086/518564 PMID: 17503334

17. Guan Y, Stephens M. Practical Issues in Imputation-Based Association Mapping. PLoS Genet. 2008 12; 4(12):e1000279. doi: 10.1371/journal.pgen.1000279 PMID: 19057666

18. Marchini J, Howie B. Genotype imputation for genome-wide association studies. Nature Reviews Genetics. 2010; 11(7):499–511. doi: 10.1038/nrg2796 PMID: 20517342

19. Long JC. The Genetic Structure of Admixed Populations. Genetics. 1991 02; 127(2):417–428. Available from: http://www.ncbi.nlm.nih.gov/pmc/articles/PMC1204369/. PMID: 2004712

20. Bhatia G, Tandon A, Patterson N, Aldrich M, Ambrosone C, Amos C, et al. Genome-wide Scan of 29,141 African Americans Finds No Evidence of Directional Selection since Admixture. The American

Journal of Human Genetics. 2015 April; 95(4):437–444. Available from: http://www.cell.com/ajhg/abstract/S0002-9297(14)00355-3. doi: 10.1016/j.ajhg.2014.08.011

21. Bersaglieri T, Sabeti PC, Patterson N, Vanderploeg T, Schaffner SF, Drake JA, et al. Genetic Signatures of Strong Recent Positive Selection at the Lactase Gene. Am J Hum Genet. 2004 Jun; 74 (6):1111–1120. Available from: http://www.ncbi.nlm.nih.gov/pmc/articles/PMC1182075/. doi: 10.1086/421051 PMID: 15114531

22. Li WH. The first arrival time and mean age of a deleterious mutant gene in a finite population. Am J Hum Genet. 1975 May; 27(3):274–286. Available from: http://www.ncbi.nlm.nih.gov/pmc/articles/PMC1762880/. PMID: 803010

23. Guan Y, Stephens M. Bayesian variable selection regression for genome-wide association studies, and other large-scale problems. Annals of Applied Statistics. 2011; 5(3):1780–1815. doi: 10.1214/11-AOAS455

24. Schaffner SF, Foo C, Gabriel S, Reich D, Daly MJ, Altshuler D. Calibrating a coalescent simulation of human genome sequence variation. Genome Research. 2005; 15(11):1576–1583. Available from: http://genome.cshlp.org/content/15/11/1576.abstract. doi: 10.1101/gr.3709305 PMID: 16251467

25. Baran Y, Pasaniuc B, Sankararaman S, Torgerson DG, Gignoux C, Eng C, et al. Fast and accurate inference of local ancestry in Latino populations. Bioinformatics. 2012; 28(10):1359–1367. Available from: http://bioinformatics.oxfordjournals.org/content/28/10/1359.abstract. doi: 10.1093/bioinformatics/bts144 PMID: 22495753

26. Maples BK, Gravel S, Kenny EE, Bustamante CD. RFMix: A Discriminative Modeling Approach for Rapid and Robust Local-Ancestry Inference. American Journal of Human Genetics. 2013 08; 93 (2):278–288. Available from: http://www.ncbi.nlm.nih.gov/pmc/articles/PMC3738819/. doi: 10.1016/j.ajhg.2013.06.020 PMID: 23910464

27. Churchhouse C, Marchini J. Multiway Admixture Deconvolution Using Phased or Unphased Ancestral Panels. Genetic Epidemiology. 2013; 37(1):1–12. doi: 10.1002/gepi.21692 PMID: 23136122

28. Consortium TGP. An integrated map of genetic variation from 1,092 human genomes. Nature. 2012 11; 491(7422):56–65. Available from: http://www.ncbi.nlm.nih.gov/pmc/articles/PMC3498066/. doi: 10.1038/nature11632

29. Delaneau O, Marchini J, Zagury JF. A linear complexity phasing method for thousands of genomes. Nat Meth. 2012; 9(1):179–181. Available from: http://www.nature.com/nmeth/journal/v9/n2/abs/nmeth.1785.html.

30. Acuna-Soto R, Stahle DW, Cleaveland MK, Therrell MD. Megadrought and Megadeath in 16th Century Mexico. Emerging Infectious Disease [serial on the Internet]. 2002; 8(4). Available from: http://wwwnc.cdc.gov/eid/article/8/4/01-0175.

31. Marr JS, Kiracofe JB. Was the huey cocoliztli a haemorrhagic fever? Medical History. 2000 07; 44 (3):341–362. Available from: http://www.ncbi.nlm.nih.gov/pmc/articles/PMC1044288/. PMID: 10954969

32. Acuna-Soto R, Romero LC, Maguire JH. Large epidemics of hemorrhagic fevers in Mexico 1545-1815. The American Journal of Tropical Medicine and Hygiene. 2000; 62(6):733–9. Available from: http://www.ajtmh.org/content/62/6/733.abstract. PMID: 11304065

33. Hellenthal G, Busby GBJ, Band G, Wilson JF, Capelli C, Falush D, et al. A Genetic Atlas of Human Admixture History. Science. 2014; 343(6172):747–751. Available from: http://www.sciencemag.org/content/343/6172/747.abstract. doi: 10.1126/science.1243518 PMID: 24531965

34. Xu H, Guan Y. Detecting Local Haplotype Sharing and Haplotype Association. Genetics. 2014; 197 (3):823–838. doi: 10.1534/genetics.114.164814 PMID: 24812308

35. Shriner D, Adeyemo A, Ramos E, Chen G, Rotimi C. Mapping of disease-associated variants in admixed populations. Genome Biology. 2011; 12(5):223. doi: 10.1186/gb-2011-12-5-223 PMID: 21635713
